# Supporting Zoo Asian Elephant (*Elephas maximus*) Welfare and Herd Dynamics with a More Complex and Expanded Habitat

**DOI:** 10.3390/ani11092566

**Published:** 2021-08-31

**Authors:** Sharon S. Glaeser, David Shepherdson, Karen Lewis, Natalia Prado, Janine L. Brown, Bob Lee, Nadja Wielebnowski

**Affiliations:** 1 Oregon Zoo, 4001 SW Canyon Road, Portland, OR 97221, USA; shepherdsond@gmail.com (D.S.); karend.lewis@gmail.com (K.L.); blee@cabq.gov (B.L.); Nadja.Wielebnowski@oregonzoo.org (N.W.); 2 Center for Species Survival, Smithsonian Conservation Biology Institute, Smithsonian National Zoological Park, Front Royal, VA 22630, USA; PradoN@si.edu (N.P.); BrownJan@si.edu (J.L.B.); 3 Department of Biology, Adelphi University, Garden City, NY 11530, USA; 4 ABQ BioPark, 903 10th St. SW, Albuquerque, NM 87102, USA

**Keywords:** welfare, behavior, sociality, locomotion, GPS, glucocorticoids, adrenal activity, habitat evaluation, feeding predictability, elephant

## Abstract

**Simple Summary:**

Ensuring good health and welfare is an increasingly important consideration for conservation of endangered species, whether free-ranging or managed to varying degrees under human care. The welfare-based design of a new habitat for Asian elephants focused on meeting the elephants’ physical, physiological, psychological, and social needs 24 h a day and across life stages. In this study, multiple elephant health and welfare indicators measured throughout transition and acclimation from the previous habitat to this new habitat provided evidence that the complexity, flexibility, space, and resource distribution of the new habitat was effective in improving overall welfare. The elephants were more active and walked farther on a daily basis in the new habitat, with an average walking distance of over 15 km per day. Disbursement of food with less temporal and spatial predictability increased foraging opportunities, which is important for psychological well-being of this species. All individuals showed adaptive and normal adrenal responses to the changes and challenges throughout the construction period and subsequent acclimation to a novel environment. They demonstrated social dynamics of a healthy herd in both habitats with transitions of individuals through life stages; however, in the new habitat they exhibited more autonomy in choosing whom to associate with socially, suggesting increased social equity for individuals.

**Abstract:**

Ensuring good health and welfare is an increasingly important consideration for conservation of endangered species, whether free-ranging or managed to varying degrees under human care. The welfare-based design of a new habitat for Asian elephants at the Oregon Zoo focused on meeting the elephants’ physical, physiological, psychological, and social needs 24 h a day and across life stages. The habitat was designed to encourage activity, promote species-typical behaviors, support changing social dynamics, offer increased opportunities for choice, and provide biologically meaningful challenges. In this 4-year study, we monitored elephant health and welfare indicators throughout the transition and acclimation from the previous habitat to the new habitat. Several welfare indicators obtained through longitudinal hormone analyses, behavior assessments, and GPS measurement of walking distance and space use provided evidence that these goals were achieved. The elephants were more active and walked farther on a daily basis in the new habitat, with an average walking distance of over 15 km per day. A switch from primarily caretaker-delivered food to seeking food on their own indicates that the disbursement of food with less temporal and spatial predictability increased foraging opportunities, which better satisfies appetitive motivations important for psychological well-being. All individuals showed adaptive and normal adrenal responses to change and challenge, with the highest fecal glucocorticoid metabolite (FGM) concentrations and variability during the construction phase, and a return to previous baseline concentrations in the new habitat, suggesting they acclimated well to the new environment. The elephants expressed a diverse range of species-typical behaviors and demonstrated social dynamics of a healthy herd in both habitats with transitions of individuals through life stages. They exhibited more autonomy in choosing whom to associate with socially and also by choosing different aspects of their environment with regular indoor/outdoor access and extensive resource use in the new habitat. Findings indicate that the complexity and flexibility of the new habitat and habitat management has been effective in improving overall welfare by providing meaningful challenges and the opportunity to express appetitive behaviors, by offering choice in environmental conditions, and by providing the space and resource distribution to support evolving herd dynamics and increased social equity for individuals.

## 1. Introduction

Ensuring good health and welfare is an increasingly important consideration for conservation of endangered species, whether free-ranging or managed to varying degrees under human care. As zoos around the world more extensively use evidence-based approaches to manage animals for improved welfare and conservation contributions [1,2,3,4,5,6,7], there is an increasing need to identify environmental factors that contribute to welfare outcomes. Within the scientific and legislative communities, definitions of animal welfare frequently reference overlapping conceptions of welfare: health or biological functioning, emotional or affective states, and the naturalness of an animal’s life [8]. Animal welfare, as defined by the Association of Zoos and Aquariums, refers to an animal’s collective physical and mental states over a period of time, and is measured on a continuum from good to poor [3]. The Oregon Zoo took a welfare-based approach in the design of its new elephant habitat—an approach focused on meeting the elephants’ physical, physiological, psychological, and social needs 24 h a day and across life stages. The habitat was designed to encourage activity, promote species-typical behaviors, support evolving social dynamics, offer increased opportunities for choice, provide biologically meaningful challenges, and exercise cognitive abilities. Design decisions were informed by decades of research, hands-on experience, and an understanding of elephants’ individual needs, and were ultimately supported by outcomes from the largest zoo-based elephant welfare study to date [9], which found a variety of social, housing, and management factors associated with multiple indicators of health and welfare in both Asian and African elephants [10,11,12,13].

To accomplish the Zoo’s elephant care goals, the habitat needed to support natural herd dynamics—to live in multi-generational matrilineal groups in which bulls could join or leave as they would in free-ranging populations [14], and for young males to have the opportunity to learn from older bulls [15,16,17,18,19]. It required a flexible space with indoor-outdoor access, an increased ability for elephants to choose where and how they spent their time and who they spent it with, and an enriched environment with a variety of features to engage with. The importance of providing animals with appropriate challenges [20,21] and a balance between choice and control [22,23,24,25] through environmental enrichment [26,27,28,29] has been convincingly documented for a wide variety of species, including elephants [30].

The habitat was designed to support flexible feeding strategies to encourage foraging and exploration, with unpredictability of food disbursement across time and space to help fulfill the elephants’ occupational need to forage. A diversity of food-delivery methods was used to provide foraging and feeding opportunities 14 to 16 h per day, including crepuscular and nocturnal periods to more closely mimic the foraging patterns of free-ranging elephants [17,31] and support a 24/7 approach to animal welfare [32]. Timed feeders were dispersed throughout the habitat and released food at programmable intervals, overhead feeders required elephants to stretch and sometimes climb, herd feeders required reaching down, and other puzzle feeders demanded manipulation to acquire food. The behaviors associated with finding food can be broken down into three stages: seeking food, acquiring food, and consuming food [33]. Veasey [33] defines “foraging” as the seeking of opportunities to acquire food, which encompasses multiple behaviors and cognitive processes, including walking, sensing and information gathering, decision making, learning, problem solving, and socializing [34]. Studies have shown that providing food in a way that promotes exploratory behavior can reduce levels of stereotypic behaviors (reviewed in [26]). Varying food predictability can promote foraging in captive animals, with the appropriate degree of predictability depending on the feeding ecology of the species [35]. In the wild, Asian elephants inhabit a wide range of habitats from rainforest (a predominantly browse-dominated habitat), to savanna (a predominantly grass dominated habitat), and their feeding ecology includes both browsing and grazing (reviewed in [34]), moving between food patches or moving through a space continuously feeding, and using a range of foraging skills to locate and access food [17,31]. Finally, the shape and topographical complexity of the habitat was designed to encourage exploration and activity; the elephants could not see the entire space from any one vantage point and so got exercise simply by maneuvering through it. The hilly terrain, climbing features, and varied surfaces—including deep sand and hills of dirt—provided stimulation and physical challenges.

Indicators of welfare pre- and post-occupancy can provide insight into the effectiveness of habitat design and features. Central to the welfare framework is that welfare indicators are quantitative measurements that include both positive and negative affective states [36]. Whitham and Wielebnowski [36] recommend integrating behavioral, physiological, and biological measures of good well-being, as each measure alone can be limited in its usefulness in interpretation. Hence, in the current study, multiple indicators of positive and negative states were measured in a pre-/post-occupancy welfare assessment.

Increased walking distance in both African and Asian zoo-housed elephants has been associated with feeding diversity and unpredictable feeding schedules [37]. In free-ranging elephants, walking activity and patterns are affected by a variety of factors, including the distribution and availability of resources, season, age, sex, reproductive state, presence of calves or juveniles in a group, ranking of social groups, and human activities [14,38,39,40,41,42,43,44]. The relationship between resource availability and walking distance suggests that walking varies in response to external conditions. Under professional care, ample food and water are provided so the need for walking is reduced. However, walking also supports exploratory behavior, which has an information-gathering function and may be rewarding even when not for the purpose of resource acquisition [45,46,47]. In addition, there is evidence that daily exercise in general improves animal body condition [13] and foot health [48]. Thus, one goal of the new design was to encourage more activity, including walking.

Welfare monitoring often includes an evaluation of hormones indicating reproductive state and adrenal activity to assess physiological aspects of animal health and well-being [49,50,51]. Indicators of stress are commonly measured as welfare outcomes, with the most frequently used indicator being glucocorticoids (e.g., cortisol, corticosterone) secreted from the adrenal cortex in response to a variety of stimuli [52,53,54,55,56]. When an individual experiences a major change constituting a real or perceived stressor, an adaptive physiological response occurs that includes resilience, whereby glucocorticoids secreted from the adrenal cortex are temporarily elevated and then subsequently decrease back to a baseline. Because the stimuli that activate the stress response can be positive or negative, it is important to measure additional indicators of welfare, such as reproductive, behavior, and health parameters.

Behavior measurements provide information on activity level, social relationships, habitat utilization, resource use, and the choices animals make. Expression of species-typical behaviors at appropriate rates is a potential indicator that the needs of an animal are being met and that it has good health and well-being [57], and activity budgets of zoo animals are increasingly being used as they relate to both physical and psychological welfare. By monitoring the behavior of groups or individuals across different conditions, we can identify factors associated with behavioral change as well as behavioral patterns that are persistent [58]. Individuals respond differently to the same stimuli, and a facility transition for this group of elephants was expected to present many opportunities and challenges. Activity budgets can provide a baseline against which to assess the response to change. Social interactions tell us about relationships and choices given social opportunities; in long-lived social species, like elephants, it is particularly important to support and monitor evolving herd dynamics across life stages. Engagement with resources informs us on how various aspects of the physical environment support self-maintenance and provide opportunities to overcome appropriate challenges, to make choices, and allow individuals some level of control over their environment (reviewed in [36]).

Given the significant changes to the Oregon Zoo exhibit, a 4-year study was conducted to evaluate the effectiveness of the new design in achieving elephant care goals through assessment of welfare indicators during occupancy in the previous habitat, through construction, and 1 year after transition to the new habitat. Indicators included distance walked through GPS monitoring, reproductive and adrenal hormone analyses, and detailed behavior assessments. By comparing the distance walked and patterns of space use in different habitats, we can better understand the social, environmental, and management-related factors influencing movement, and evaluate walking distance in association with other indicators of welfare. Through hormone monitoring, we aimed to determine how the elephants dealt with the challenges of construction and transition to new environments, if their responses had any long-term negative effects, or perhaps if there were beneficial effects of arousal and the opportunity to exercise cognitive and behavioral skills [20]. Behavioral assessments allowed us to evaluate evolving social dynamics and assess habitat-related factors associated with activity budgets, activity levels, and opportunities for choice. The captive environment offers the ability to collect routine data on individuals with known health status and life histories [59]. Using quantitative behavioral and physiological data to evaluate welfare outcomes on individual elephants under multiple environmental conditions, we were able to test the direct influence of various factors on welfare and assess the effectiveness of habitat design features and associated management practices in achieving optimum welfare.

Outcomes of this study provide insight into how habitat features help meet the physical, physiological, psychological, and social needs of elephants under human care. Wildlife populations are facing a changing landscape and coming under varying degrees of human management. Our assessment methods can provide a model for long-term monitoring of well-being across life stages and also for future investigations on the impact of various changes on wildlife with relevance to both in situ and ex situ conservation—facility transfers, habitat modifications, changes in social structure, rescue, rehabilitation, release, transition to captivity, translocations, habitat conversion, resource extraction, introduction of tourism, and other anthropogenic disturbances.

## 2. Materials and Methods

### 2.1. Animals

The Asian elephants (n = 5) included in this study were housed at the Oregon Zoo (females: n = 3 *Elephas maximus indicus*, n = 1 *E. m. borneensis*, males: n = 1 *E. m. indicus*) (Table 1) as a herd. Female 3 was the mother of M-juvenile and F-calf; Female 1 and Female 3 were related with the same grandfather; Female 2 was wild-born and unrelated. Three adult bulls were also housed at the zoo but were not included in the study because of medical conditions. This study was approved by the Welfare and Research Review Committees at the Oregon Zoo.

### 2.2. Study Timeline

**Figure 1:**
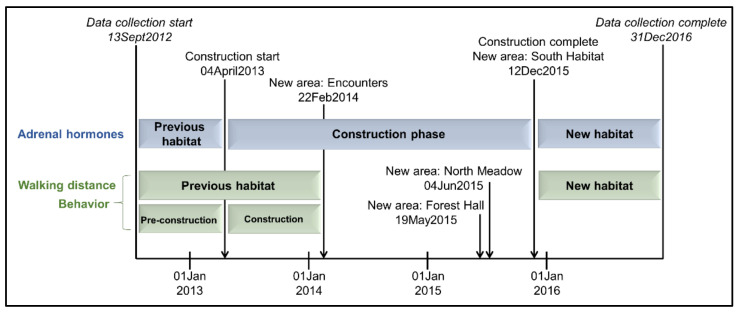
Study timeline showing habitat phases and associated data analyses.

The timeline comprised three habitat phases: occupancy in the previous habitat; period of change with construction, introduction to new spaces (four new areas), reconfigurations of familiar spaces, and a move to the new building; and occupancy in the new habitat. Sample collection began in September 2012 while the elephants were in the original habitat, and continued through the end of 2016, 1 year after full opening of the new habitat. The timeline of habitat changes is illustrated in Figure 1.

For analysis of adrenal hormones, the habitat phases were defined by construction start and end dates, resulting in durations of 29 weeks in the previous habitat, 140 weeks during the construction phase, and 55 weeks in the new habitat. For analysis of behavior and walking distance, the first introduction to a new yard on 22 February 2014 marked the end of the previous habitat phase, and the first day with full access to the new habitat on 12 December 2015 marked the start of the new habitat phase, resulting in durations of 75 weeks in the previous habitat and 55 weeks in the new habitat. The previous habitat phase was further divided into pre-construction (29 weeks) and construction (46 weeks) to assess the impact of construction activities alone using behavioral methods.

### 2.3. Habitat Descriptions

The previous habitat consisted of two oval-shaped outside yards on either side of the elephant building, both with sand substrate. The smaller yard was 1050 m^2^, with a pool and shade structure. The larger yard was 2350 m^2^, with a 302,800-L pool surrounded by a concrete apron, a shade structure, a scratching wall mid-yard with hardware for attaching enrichment objects, a vertical log for hanging enrichment, a large water drink tank, and one timed feeder. The inside area consisted of seven temperature-controlled rooms with rubber flooring [60], each with a water tank and several structures that provided options for enrichment. With two yards, outside access could be given to two groups of elephants (2 groups, 1 group and 1 individual managed as a solitary animal (adult bull), or 2 individuals) at a given time. The door from the larger yard opened into pass-through areas rather than rooms, so extended indoor/outdoor access to the larger yard was not possible. Three doors from the smaller yard opened into inside rooms, so indoor/outdoor could be provided for one group or individual at a given time.

The new habitat is approximately four times the size of the previous habitat, with a total habitat area of 18,210 m^2^. Three connected outdoor yards have varied terrain and a perimeter of a 2.1 km. A separate acclimation area includes an outside yard and inside room. Indoor spaces total nearly 3065 m^2^. All elephant habitat areas are covered with a sand substrate and also with dirt in some areas outside. Outdoor and indoor spaces are linked with multiple options for configuring the space for groups and individuals. The indoor space is climate controlled, and outdoor shade structures are installed with overhead heaters and misters. Food-delivery resources include timed feeders throughout the habitat, various overhead feeders (nets, barrels, puzzles), and partially buried herd feeders. Natural vegetation along the habitat perimeter includes trees and bamboo providing additional browsing opportunities, and grass for limited grazing. Water features include a 605,600-L pool with a depth of 3.7 m, a wading pool, water cannon, drinkers throughout the habitat, and a deluge sprinkler system indoors to simulate rain and also for deep cleaning of the sand substrate. Other features include dirt hills and mud wallows, large logs and rocks for climbing, and structures such as vertical logs that provide options for caretakers to attach non-permanent environment enrichment objects. With three outdoor yards and an acclimation yard, four groups or individuals can have outdoor access at a given time. Three doors from the largest outside yard open to the indoor spaces. Combined with the acclimation area, this allows two groups or individuals to have indoor/outdoor access at a given time.

### 2.4. Data Analysis

Statistical analyses are described in the Section 2 for each welfare indicator. Descriptive statistics and charting were performed in Excel 2016. Statistical analyses were conducted in R version 3.6.3 for Windows [61]. Significance was assessed at the *p* = 0.05 level.

### 2.5. Walking Distance and Space Use

In 2012, scientists measured outdoor walking distance in 56 adult female African and Asian elephants, 12 years and older, in 30 North American zoos accredited by the Association of Zoos and Aquariums (AZA) [37] as part of a larger multi-institutional elephant welfare study [9]. The Oregon Zoo’s elephants were part of this study, and therefore these existing data were used as the measurement of outdoor daily walking distance in the previous habitat. The methodology in Holdgate et al. [37] was implemented in this current study to compare distance walked between the new habitat and previous habitats.

Custom-fit bracelets equipped with GPS data loggers (BT-Q1000XT GPS Travel Recorder, QStarz International Co., Taipei, Taiwan) contained in a waterproof case (OtterBox Drybox OTR3-1000S, OtterBox, Fort Collins, CO, USA) were worn by the same two adult females as in the 2012 study (Female 1 and Female 2) plus two males (M-juvenile and an adult bull). The third adult female, Female 3, was not measured for distance walked as it was expected her movement would be significantly influenced by her calf [62]. Bracelets were worn for 24-h periods, approximately every 2 weeks, from June 2014 through December 2016. All elephants were acclimated to wearing the bracelets through desensitization using positive reinforcement. Nevertheless, GPS monitoring days did not coincide with behavior monitoring days to avoid any influence the GPS bracelet might have had on behavior. Data collection logs were kept to indicate the time bracelets were put on and removed, areas to which elephants had access, and whether these areas were indoors, outdoors, or had indoor/outdoor access.

**Figure 2:**
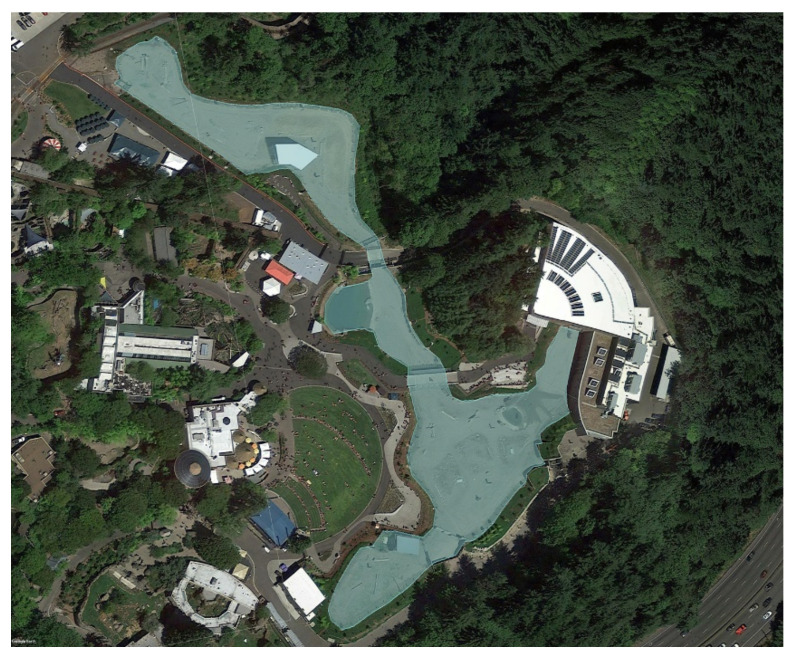
New habitat with outdoor area shaded using ArcMap.

GPS units were programmed to record data at 5-s intervals. Each location data point included the date, time, latitude, longitude, and two indices of location estimate quality (number and geometry of satellites used) [37,63]. GPS data are unreliable when communication between satellites and receivers is obstructed, for example under a dense forest canopy or inside a building; therefore, data points known to have occurred while the elephants were indoors were removed. In ArcMap software (v. 10.6, Environmental Systems Research Institute, West Redlands, CA, USA), habitat boundaries were defined using Google Earth imagery (Figure 2). GPS data were then mapped onto the habitat, and any data points that occurred indoors or drifted outside the habitat boundaries were removed using a clipping. Clipped data were exported to Excel, then data points that failed to meet location estimate quality criteria were removed using methods in Holdgate et al. [37]. The Euclidean (straight line) distance between consecutive data points was calculated, then screened for distances greater than an elephant can travel using a near maximal velocity of 6.8 m/s measured for elephants [64]. The final straight-line distance between consecutive data points was then summed to obtain total distances traveled for each day. Mann-Whitney U-tests were used to compare mean daily walking distance between the new and previous habitats.

Holdgate et al. [37] collected GPS data for 5 days within a month that minimized inter-zoo variation in predicted daily temperature (late August/early September for the Oregon Zoo). Walking distance was measured only for elephants that had outdoor access for at least 20 of the 24 h for at least 3 days. For comparison of distance walked in the new habitat to the previous habitat, a subset of the data was used that met the same criteria of 20 h and the same season (July through September) rather than limiting data collection to the same month. In this timeframe, Female 1 had 6 days that met criteria, Female 2 had 7 days, and M-juvenile had 3 days.

### 2.6. Reproductive and Adrenal Hormones

Adrenal and reproductive hormones were analyzed to assess adrenal activity and reproductive cycling throughout the study period, from September 2012 through December 2016. Fecal and blood samples were collected weekly from all individuals, and were collected in the morning to control for any diurnal patterns of hormone secretion and excretion [65].

Progestagens and testosterone in serum are routinely measured in Oregon Zoo elephants to determine ovarian cyclicity in females and musth correlates in males. All elephants in this study were trained for voluntary blood collection (without sedation) as part of their normal management routine. Blood was collected (3–9 mL) into red top serum separator tubes from an ear or leg vein by elephant care staff. Blood was maintained at ~4 °C, then centrifuged at 1500× *g* within a few hours of collection to separate serum, and stored at −20 °C or colder until analysis. Serum samples were analyzed unextracted. Progestagens and testosterone in serum samples collected through 2014 were measured in Oregon Zoo’s endocrine lab using solid-phase ^125^I radioimmunoassays (RIA) (Siemens Healthcare Diagnostics Inc.) previously validated in elephants (progesterone [66,67,68]; testosterone [69,70]). After these RIA assays were discontinued in 2014, samples from 2015 through 2016 were analyzed using double-antibody enzyme immunoassays (EIA) validated for Asian elephants (progesterone: Oregon Zoo, unpublished; testosterone: [71]). Assay sensitivities were 0.05 ng/mL for the progesterone RIA, 0.033 ng/mL for the progesterone EIA, 0.10 ng/mL for the testosterone RIA, and 0.015 ng/mL for the testosterone EIA. Values using the EIA and RIA assay for the same samples were significantly correlated (testosterone: r(49) = 0.93; progesterone: unpublished).

Glucocorticoid metabolites excreted in feces (fecal glucocorticoid metabolites, FGMs) were measured in samples collected with defecation times between 0700 h and 1100 h to minimize possible diurnal fluctuation in FGM concentrations. Samples were collected within 2 h of defecation, and only from individuals observed defecating to ensure positive identification. Fecal matter was collected from the center of the bolus(es), then placed in zip-lock bags and stored at −20 °C or colder until analysis. Fecal samples were extracted using a dry-weight shaking extraction technique adopted from Scarlata et al. [72] and modified by Brown et al. [73], with slight differences in centrifugation and sonication for this study. In brief, 0.1 g (±0.01 g) of lyophilized fecal powder was placed into 16 × 125 mm glass tubes. Five mL of 80% methanol was added, the samples were vortexed at least 10 s until well mixed, agitated using a multi-tube pulse vortexer for 30 min, and then centrifuged at 2200× *g* for 20 min. Supernatants were decanted into a second tube, and the remaining pellet was re-suspended in 5 mL of 80% methanol and extracted again. The supernatants were evaporated to dryness under forced air in a fume hood overnight. Dried extracts were reconstituted in 1 mL of 100% methanol, and the tubes were then vortexed briefly sonicated for 15 min, and dried again. Final extracts were re-constituted in buffer (1 mL, 0.149 M NaCl, 0.1 MNaPO4; with pH 7.0) and the tubes sonicated for at least 30 s to dissolve particulates. Finally, neat extracts were stored at −20 °C until analysis. The average extraction efficiency was 85.7% (range, 70.8–99.5%) based on addition of ^3^H-corticosterone (tracer) to each sample prior to extraction.

FGM concentrations were determined using a double-antibody enzyme EIA with a polyclonal rabbit anti-corticosterone antibody (CJM006) validated for elephants by Watson et al. [74] and described by Brown, et al. [73]. The inter assay coefficient of variation (CV %) for the high control was 3.0%, and the low control CV% was 2.9% (n = 31 plates); intra-assay CV was maintained <10% as all samples with duplicate CVs over 10% were reanalyzed. Assay sensitivity (based on 90% binding) was 0.14 ng/mL.

Progestagen data were charted and visually inspected to determine ovarian cyclicity status based on progestagen patterns [75,76,77]. Testosterone data were charted and musth state was determined using testosterone patterns and physical and behavioral correlates of musth following methods of Glaeser [71]. Because hormone patterns rather than strictly quantitative threshold values were used to determine reproductive state, any quantitative differences between RIA and EIA values in progestagen or testosterone concentrations did not alter the reproductive state determination.

Distributions of raw FGM data were non-normal based on the Shapiro-Wilk normality test (R package “stats”), and log-transformed data failed normality tests based on values of kurtosis and skewness (calculated in Excel). Therefore, data were analyzed with non-parametric tests performed on non-transformed raw data. Sample sizes were unequal across habitat phases due to different durations of each phase, and, unfortunately, all samples collected between February and October 2013 had to be discarded due to storage issues. Comparisons in median FGM concentrations across the three phases of the project (previous habitat, construction phase, new habitat) were made using a Kruskal-Wallis rank sum test (R package “stats”). Comparisons of variability in FGM concentration across the phases were made using a Levene’s test for equality of variance (R package “lawstat”) modified to calculate deviations from the median [78] and with structural zeros removed [79]. Post-hoc pairwise comparisons of median concentrations and variability between the project phases were made using Dunn’s Multiple Comparisons (R package “FSA”) with Benjamini-Hochberg corrections applied for multiple comparisons [80]. Although statistical power may be lower when outliers are present [81], outliers were not removed for analysis because these data are biologically relevant.

### 2.7. Behavior and Resource Use

Behaviors were coded using video recordings rather than live observations to facilitate more detailed behavior assessments. Behavior data collection was designed to capture diurnal activity for both morning and afternoon periods on a weekly basis. Video was recorded every other week, on alternating Saturdays and Mondays, during two 2-h time periods (1000–1200 h and 1400–1600 h). Focal animals were recorded only from visitor viewing areas and were recorded in the order seen, left to right, to eliminate recording bias. Each focal animal was recorded for 2 min once every 30 min, beginning at the start of the hour and half hour, for a total of eight 2-min recordings per day. Video was recorded only for self-initiated behavior to exclude behaviors directed or modified by caretakers. Actual breeding was not recorded because the opportunity to breed was limited to a subset of elephants. Video was recorded by a team of 14 Oregon Zoo volunteers trained on a protocol that ensured consistency between each phase of the project, and the PI reviewed raw video and conducted periodic training updates and in-person checks to ensure adherence to protocol.

A multi-level ethogram was designed to be widely applicable. Behavior categories were defined at a high/overview level for long-term monitoring of activity budgets (Table 2). Categories were mutually exclusive and hierarchically organized by social behaviors, resource-related behaviors, then movement-related behaviors. Some categories were more granular and were broken down into specific behaviors and modifiers for more detailed assessments (Appendix A). Social behaviors organized by social function (affiliative, agonistic, arousal, reproductive, dam-calf) and with social partner modifiers were used to evaluate social dynamics. Habitat resource modifiers for interactions with the environment were used to evaluate habitat and resource use. The ethogram was constructed using published sources [10,82,83,84] and unpublished studies at the Oregon Zoo, and included both state and event behaviors. Behaviors were described objectively (i.e., without interpretation of intent or purpose) such that they could be recognized by multiple observers trained in behavior methodology but without expertise in elephant behavior.

Social interactions were assigned the highest priority because social factors play an important role in the behavior and ecology of wild elephants, and proper social management can be used to promote positive welfare. Rare and unexpected social behaviors were also included in the event of a major change in social structure (e.g., transfer in of an elephant). Although social interactions are mediated by various forms of communication (reviewed in [82]), video observation methods did not allow inclusion of acoustic communication, and olfactory communication was limited to behaviors involving the trunk that were easy to discern in video and also serve a function in assessing reproductive state [85,86]. Resource-related behaviors encompassed interactions with the physical environment and acquiring nutrition, which were important in evaluating efficacy of the new habitat. Although aspects of the environment may be enriching, food-based enrichment and habitat features were not classified as enrichment in this study; rather, enrichment was defined as non-food objects added to the environment by care staff which adhered to goals of providing stimuli and eliciting species-appropriate behaviors for the benefit of the animals [27,87]. Behaviors for acquisition of nutrition included feeding, defined as the acquisition and consumption of food, and food object interaction as an unequivocal indicator of food searching or foraging. Although locomotion is a component of foraging behavior [33], locomotion also occurs in contexts outside of foraging and was considered as an independent behavior in this study.

Behaviors were coded from video in Observer XT 11.5 (Noldus Information Technology) by the PI and observers trained to meet 85% inter-observer reliability criteria. Behaviors were recorded using instantaneous focal animal sampling [88], with a sampling interval of 30 s. The sampling interval was determined using a method modified from Margulis and Westhus [89], whereby behaviors were scored at 30 s then subsampled to lengthen the interscan interval, then rates of behaviors were compared. At every sampling interval, observers coded caretaker presence to ensure only self-initiated behaviors were included in the analysis, proximity of focal to other elephants, and a single behavior within the top-most behavior category that applied, for a total of four behaviors per 2-min observation. Social behaviors that required interpretation to categorize correctly as affiliative, agonistic, or reproductive (e.g., playing vs. sparring, rump present as submissive vs. reproductive context) were reviewed by the PI. Coding was conducted over a period of approximately 2 years, so interim reliability tests were conducted to ensure criteria were maintained. Interim tests using 20 observations with five comparisons each resulted in an average of 89% inter-reliability for all observers (range 85% to 92%), which equated to a pooled Cohen’s kappa of 0.82 and a confidence interval of 0.72 to 0.91.

Observation data were exported to Excel for charting and analysis in R. Behavior of the focal animal was visible in 86% of the samples (i.e., not visible 14%), and only visible samples were included in analysis. Behavior categories were not independent of each other because they were mutually exclusive; therefore a G-test of independence [90,91] in the R package “DecTools” [92] was used to investigate changes in activity budgets between the new and previous habitats for each elephant and all elephants combined. Within behavior categories, the frequencies of recorded behavior were not normally distributed based on the Shapiro-Wilk test; therefore, a non-parametric Mann-Whitney U-test was used for all pairwise comparisons of behavior in the new versus previous habitats. The occurrences of each behavior and each modifier (for social partner and habitat resource) were tallied to calculate percentages of visible samples engaging in behaviors, interacting with each herd mate, and interacting with each habitat resource. Comparisons were made only for those behavioral categories that occurred at rates greater than 2% in at least one habitat.

## 3. Results

### 3.1. Walking Distance

**Figure 3:**
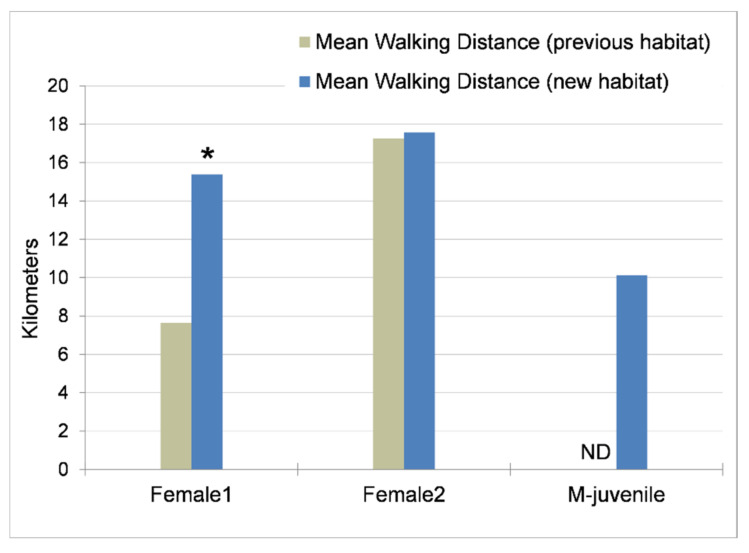
Mean daily walking distance comparing the previous habitat and new habitat. Asterisk (*) above bars indicates statistically significant differences between habitats. ND indicates no data.

Female 1 (Figure 3) doubled her mean daily walking distance from 7.6 km in the previous habitat to 15.4 km in the new habitat (W = 18, *p* = 0.024). Female 2 walked similar distances in both habitats, with a mean of 17.3 km in the previous habitat and 17.6 km in the new habitat (Figure 3). With the percentage of time spent performing repetitive behaviors (e.g., locomotor stereotypy) removed from distance calculations, Female 1 showed an increase from 7.3 km in the previous habitat to 14.7 km in the new habitat, and Female 2 showed an increase from 15.4 km to 17.1 km. Across all seasons in the new habitat, Female 1 averaged 13.8 km per day, with a maximum of 20.8 km; and Female 2 averaged 16.2 km per day, with a maximum of 23.7 km. M-juvenile had a mean daily walking distance of about 10 km per day (Figure 3), but this result underestimated his actual walking distance. Due to his destruction of various bracelet designs, data were collected on M-juvenile only from January to May in 2016, and on the days when he wore the GPS bracelet, he chose to spend most of his time indoors, resulting in a maximum of 9 h of data outdoors in a 24-h period of recording. We know that the elephants walked farther than GPS calculations indicated because movement was measured only in the outside habitats; they walked and exercised regularly in the large indoor habitat where GPS measurements were not possible.

### 3.2. Space Use

**Figure 4:**
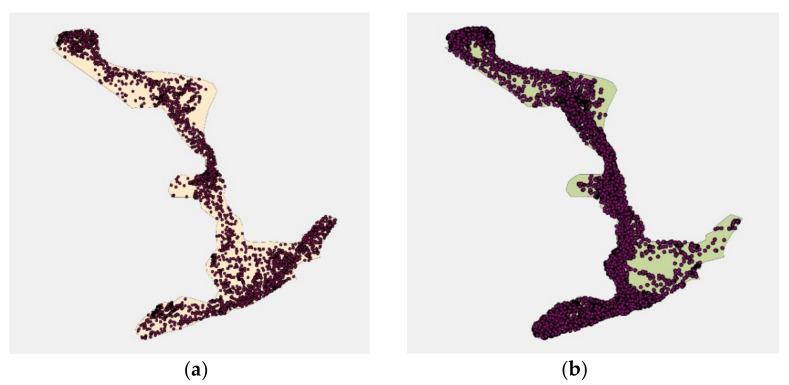

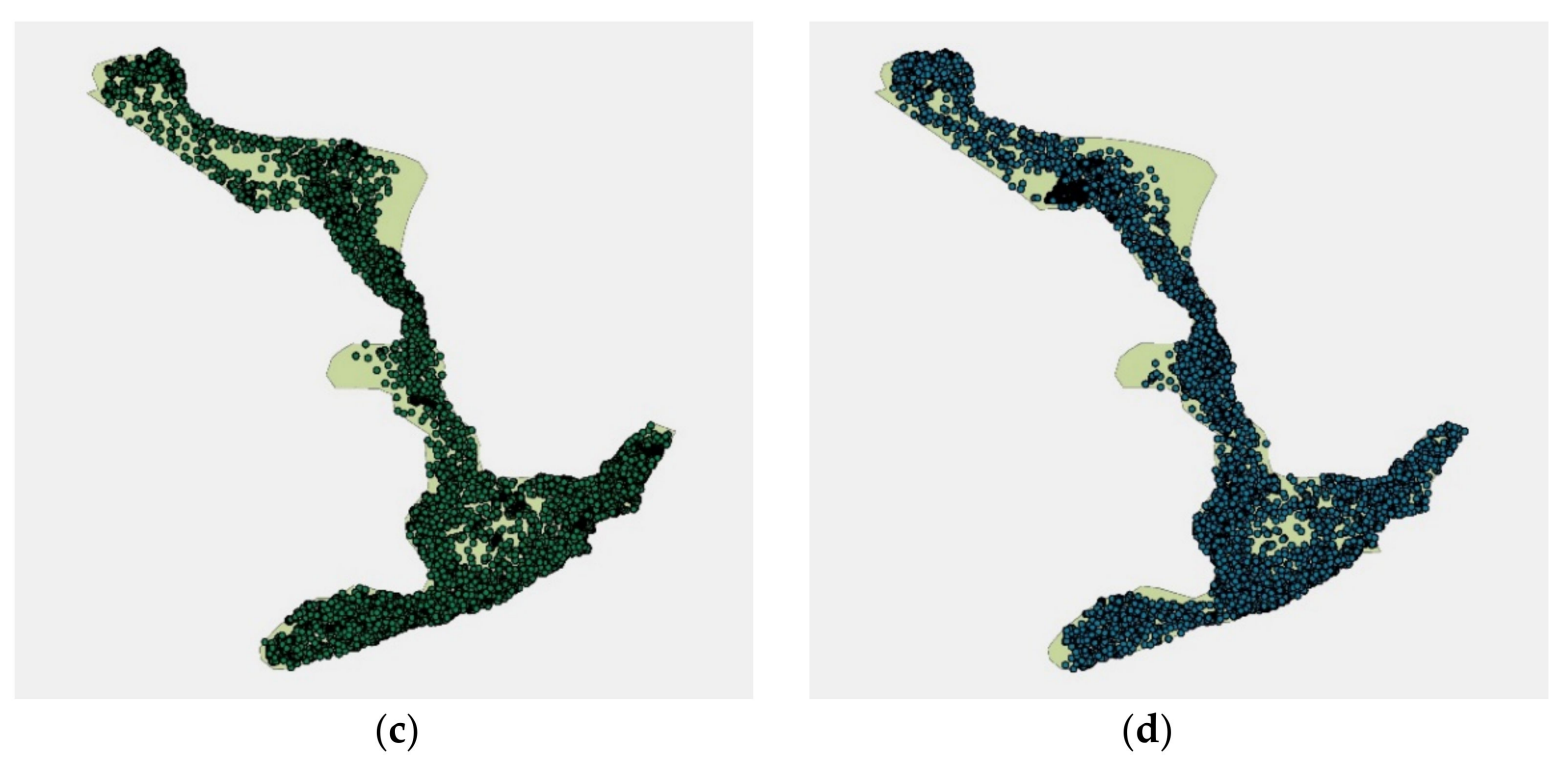
Representative examples of GPS data in outdoor areas of the new habitat at different times of year. (**a**) M-juvenile using the entire habitat including pools and dirt mounds over a 12-h period (May 2016). (**b**) Female 2 using areas nearest public viewing areas, dirt mounds, and feeders at the top of the hill in the north habitat (August 2016). (**c**) Female 1 using the entire habitat in warmer weather (August 2016). (**d**) Female 1 using the entire habitat in cooler weather (November 2016).

GPS mapping (Figure 4) shows GPS data points where elephants walked in the outdoor portion of the new habitat. Visual inspection of all data showed they were using all habitat areas rather than concentrating their use in a few preferred areas or avoiding certain areas. Similar usage patterns were observed across seasons, despite extreme differences in temperature and weather, and fluctuations in the number of zoo visitor and special events. The space-use patterns clearly suggest this is the result of the more dispersed and diverse foraging and feeding opportunities and the more enriched environment. Movement in the new habitat was more under the control of the elephants than in the previous habitat. The design of the previous habitat was more limiting in how many elephants or groups could have indoor/outside access at the same time, requiring care staff to keep some individuals inside in order to give others outside access. In the new habitat, elephants had outdoor access for more than 20 h a day in every month of the year except January, and individuals chose to spend anywhere from 6 to 20 h outdoors.

### 3.3. Reproductive and Adrenal Hormones

All females continued to show regular ovarian cycles throughout the construction phase and in the new habitat. M-juvenile exhibited signs of his first musth with temporal gland secretions coinciding with elevated testosterone at age 6 years, during the construction phase.

**Figure 5:**
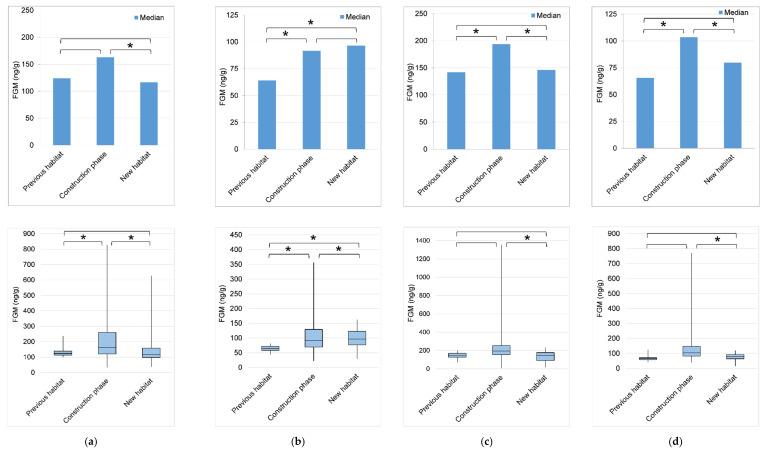
Comparison of median and variability (inter-quartile range and range) fecal glucocorticoid metabolite (FGM) concentrations between the previous habitat, the construction phase, and the first year in the new habitat. (**a**) Female 1; (**b**) Female 2; (**c**) Female 3; (**d**) M-juvenile. Asterisk (*) indicates a statistically significant difference between habitat phases.

All elephants showed significant differences in FGM median concentrations and variability across habitat phases, with the highest concentrations and greatest interquartile range (IQR) during the construction phase, and lower concentrations and IQR in the previous and/or the new habitats (Figure 5, Table 3, Table 4 and Table 5).

Female 1 (Figure 5a) showed higher median FGM concentrations in the construction phase compared to the new habitat, a higher but not significantly different concentration in the construction phase compared to the previous habitat, and similar concentrations between the new and previous habitats (Table 4). Variability was highest in the construction phase compared to previous and new habitats (Table 5). Female 3 (Figure 5c) and M-juvenile (Figure 5d) showed higher median FGM concentrations in the construction phase compared to the previous and new habitats. Concentrations were similar between the new and previous habitats (Table 4). Variability was highest in the construction phase, but differed significantly only compared to the new habitat, with no significant difference between the previous and new habitats (Table 5). Female 2 (Figure 5b) exhibited a somewhat different pattern, showing continued elevated FGM concentration in the new habitat. However, she did show a decline in FGM concentrations immediately following introductions to new habitat areas. Variability was highest in the construction phase, followed by the new habitat, with lowest variability in the previous habitat (Table 5).

F-calf was born only months prior to the start of construction and therefore FGMs were compared only between the construction phase and the new habitat. As with Female 1, Female 3, and M-juvenile, she showed higher median and greater variability in FGM concentrations during the construction phase (Table 4 and Table 5). This study spanned ages 1 to 4 years for F-calf, with the construction phase encompassing ages 1 to 3 years. It is not known how adrenal activity changes during early development in elephants; however, her concentrations showed intermediate levels compared to the other elephants (Table 3).

### 3.4. Behavior

#### 3.4.1. Activity Budgets

**Figure 6:**
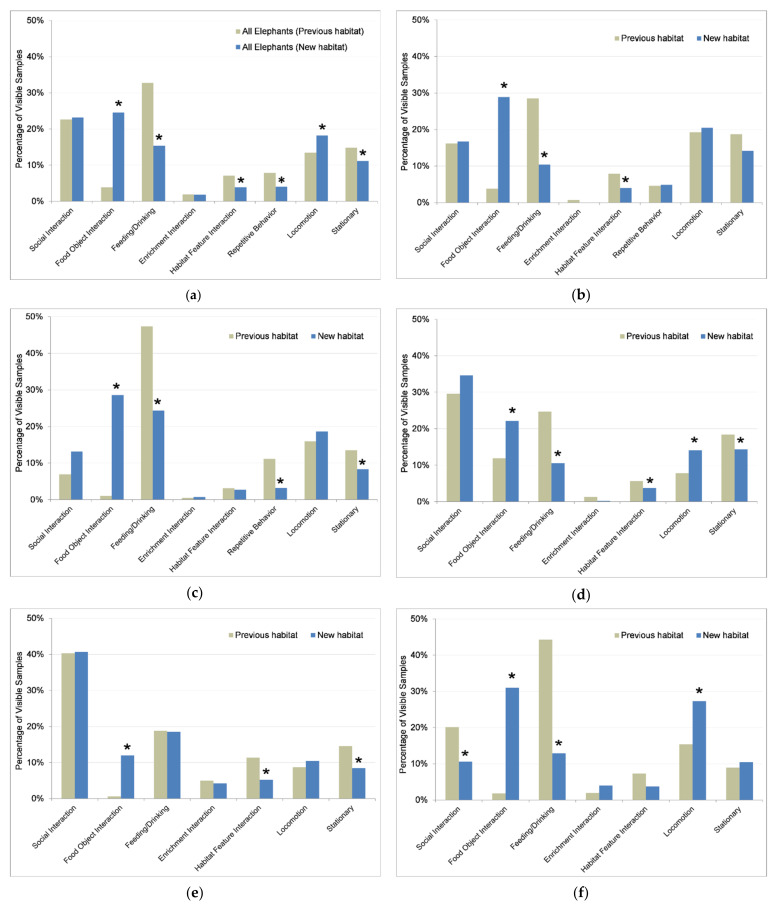
Activity budgets. Percentage of observations for each behavioral category, comparing the previous habitat and new habitat for all elephants combined and for individual elephants. (**a**) All elephants; (**b**) Female 1; (**c**) Female 2; (**d**) Female 3; (**e**) F-calf; (**f**) M-juvenile. Asterisk (*) indicates statistically a significant difference between habitats when comparing the same behavioral category.

With all elephants combined, elephants spent the majority of their daytime activity in the new habitat seeking food (i.e., interacting with food-delivery resources) and feeding on their own (39.9%), socializing and interacting with their environment in a social context (23.4%), and locomoting (18.1%), with a smaller portion of their time spent interacting with habitat features and non-food-based enrichment objects in a non-social context, e.g., in the pool alone (5.7%) (Figure 6a). Activity budgets differed between the new (n = 4107 visible samples) and previous (n = 5609 visible samples) habitats for all elephants (N = 5) combined (G-test of independence: G = 1265.80, df = 7, *p* < 0.001) and for each individual (Female 1: G = 331.11, df = 7, *p* < 0.001; Female 2: G = 501.03, df = 7, *p* < 0.001; Female 3: G = 133.08, df = 7, *p* < 0.001; F-calf: G = 151.29, df = 7, *p* < 0.001; M-juvenile: G = 544.93, df = 7, *p* < 0.001).

The average percentage of time spent foraging (seeking food) and feeding on their own during staffed hours was similar between the new (39.9%) and previous (36.6%) habitat; however, interacting with resources that disburse food (e.g., timed feeders, overhead hay nets) increased (W = 1,267,680, *p* < 0.001) six-fold in the new habitat (from 3.8% to 24.5%), while feeding without interaction with a food delivery resource decreased (W = 788,753, *p* < 0.001) by half (from 32.7% to 15.3%) (Figure 6a). Sharing of food resources (i.e., simultaneously interacting with food-delivery object or feeding from the same food source) comprised a greater percentage of the activity budget in the new habitat, increasing from 5.4% in the previous habitat to 13.9% in the new habitat, resulting in a total foraging and feeding time, either on their own or in a social context, of 42.4% in the previous habitat and 53.9% in the new habitat. Sharing of food resources also comprised a greater percentage of all social interactions in the new habitat, increasing from 24.7% in the previous habitat to 59.5% in the new habitat.

Locomotion increased (W = 1,072,717, *p* < 0.001) from an average of 13.4% to 18.2% in the new habitat, while stationary behaviors decreased (W = 921,102, *p* < 0.001) from 14.8% to 11.2%. Repetitive behaviors were exhibited by two adult females, both in the form of route tracing/pacing. They showed a decrease (W = 976,337, *p* < 0.001) in the new habitat, with average route tracing/pacing declining from 7.9% in the previous habitat to 4.0% in the new habitat. Habitat feature interaction decreased (W = 936,767, *p* < 0.001) from an average of 7.1% in the previous habitat to 3.9% in the new habitat.

The percentage of time engaged in the sum of active behaviors (social behaviors, interaction with food-delivery and non-food enrichment objects and habitat features, feeding/drinking, and locomotion) ranged from 80.0% to 85.1% across individuals in the previous habitat and 85.3% to 91.1% in the new habitat, increasing for all elephants combined (W = 1,090,275, *p* < 0.001) and for three of four females (Female 1, Female 2, and F-calf).

#### 3.4.2. Behavior of Individuals

Female 1 (Figure 6b) switched to foraging and feeding primarily from food-delivery resources in the new habitat (increased from 3.8% to 28.9%, W = 53,405, *p* < 0.001), but the switch to more independent feeding took longer than it did for the other elephants. During the first several weeks, Female 1 spent a substantial amount of time near gates, assumed to be in anticipation of caretakers delivering food rather than seeking food on her own. Caretakers attempted to “teach” her to eat out of the feeders by calling her to the feeders, which may have unintentionally prolonged the transition by perpetuating the association of care staff with food. She showed few repetitive behaviors (less than 5% of her time) in the previous and new habitat, and such behaviors occurred in infrequent bouts. She showed a notable decrease in bouts of repetitive behavior near gates as she began seeking food on her own; however, there was no significant difference between the habitats in overall time spent engaging in repetitive behaviors. Habitat feature interaction decreased (W = 38,069, *p* = 0.007) from 7.9% in the previous habitat to 4.0% in the new habitat. Time spent engaging in active behaviors increased (W = 44,935, *p* = 0.041) from 80.0% in the previous habitat to 85.3% in the new habitat. In contrast to the GPS monitoring results for this female, behavior results indicated that the amount of time spent in locomotion was not significantly different between the two habitats.

Female 2 (Figure 6c) spent almost no time (<2%) interacting with food-delivery resources in the previous habitat, whereas she spent 28.6% of her time foraging and feeding from food-delivery resources in the new habitat. However, she still spent a larger portion (24.3%) of her time than other elephants eating food that was available without object manipulation, such as live vegetation, browse, and hay left by other elephants near food-delivery resources. Time spent engaging in active behaviors increased (W = 47,267, *p* = 0.022) from 84.3% in the previous habitat to 91.1% in the new habitat, and repetitive behaviors decreased (W = 38,667, *p* < 0.001) from 11% of her time in the previous habitat to 3% in the new habitat.

Female 3 (Figure 6d) switched to foraging and feeding primarily from food-delivery resources in the new habitat (increased from 11.9% to 22.2%, W = 48,314, *p* < 0.001); however, in the previous habitat she already spent more time manipulating food-delivery resources than other elephants, so the increase was not as large for her as for other elephants. Locomotion increased (W = 46,834, *p* = 0.002) in the new habitat from 7.8% to 14.1% (almost doubled), and time spent stationary decreased (W = 37,525, *p* = 0.007) from 18.4% to 14.3%. Female 3 showed less than 0.05% of her time in repetitive behaviors in the previous habitat, and showed no repetitive behaviors in the new habitat. Habitat feature interaction decreased (W = 39,822, *p* = 0.037) from 5.6% in the previous habitat to 3.8% in the new habitat. Time spent engaging in active behaviors was not significantly different between the habitats.

F-calf (Figure 6e) adapted most readily to the new habitat, and was usually the first elephant to venture into new habitat areas. F-calf quickly learned to seek food from a diversity of food-delivery resources in the new habitat, although she still spent a larger portion of her time feeding from food that was available without object manipulation. In both habitats, she spent a larger portion of her time interacting with habitat features than other elephants, but the percentage of time decreased (W = 30,763, *p* < 0.001) from 11% in the previous habitat to 5% in the new habitat. Her time spent engaging in active behaviors increased (W = 40,006, *p* = 0.026) from 85.1% in the previous habitat to 91.0% in the new habitat, while time spent stationary decreased (W = 32,185, *p* = 0.002) from 14.1% to 8.5%. F-calf showed no repetitive behaviors in either habitat.

M-juvenile (Figure 6f) also switched from spending almost no time (<2%) interacting with food-delivery resources in the previous habitat to seeking food and feeding from food delivery-resources in the new habitat (31.0% of his time). Locomotion increased (W = 44,005, *p* < 0.001) in the new habitat from 15.4% to 27.3%. M-juvenile was the only elephant to exhibit a decrease (W = 31,320, *p* < 0.001) in social interactions, almost halving his time from 20.1% in the previous habitat to 10.6% in the new habitat. M-juvenile showed no repetitive behaviors in either habitat.

#### 3.4.3. Social Behaviors and Social Partners

**Figure 7:**
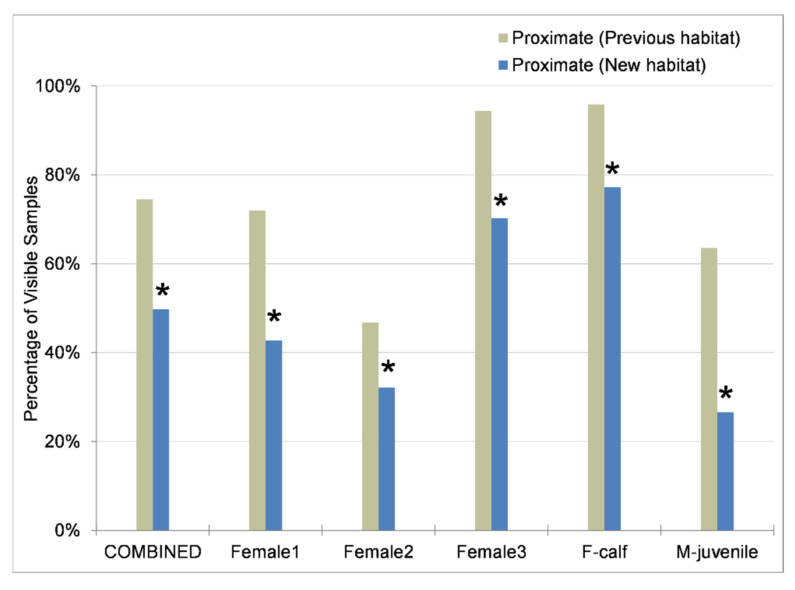
Percentage of observation samples in proximity to herdmates, comparing the previous habitat to the new habitat for individual elephants and all elephants combined. Asterisk (*) indicates a statistically significant difference between habitats.

The percentage of time spent in proximity (i.e., within 2 body lengths) of other elephants decreased (W = 790,302, *p* < 0.001) for all individuals (average 25% decrease) in the new habitat (n = 4499 samples) compared to the previous habitat (n = 5525 samples) (Figure 7). Female 3 and her calf (F-calf) had the highest percentage of time spent proximate to another elephant in both habitats, followed by Female 1. In the previous habitat, Female 2 spent the least percentage of time in close proximity to other elephants. M-juvenile showed the largest decrease (W = 23,969, *p* < 0.001) in proximity (by more than half) and was the least proximate in the new habitat.

**Figure 8:**
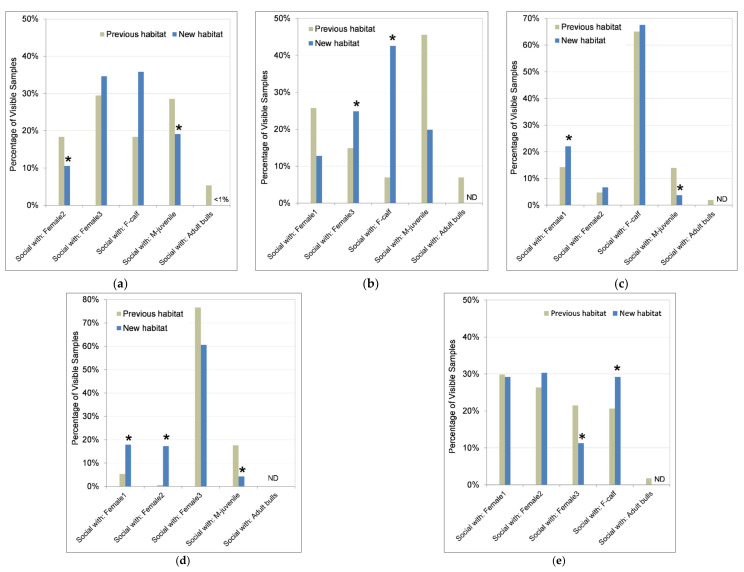
Percentage of observation samples engaging in social interactions with each herdmate: (**a**) Female 1; (**b**) Female 2; (**c**) Female 3; (**d**) F-calf; (**e**) M-juvenile. Asterisk (*) indicates a statistically significant difference between habitats. ND denotes no data with adult bulls for comparison.

Figure 8 provides insight into the social relationships among this group of elephants. Although differences in proportion of time spent engaging in active social interaction were not statistically significant except for M-juvenile (Figure 6), there were significant differences between habitats in the social partners involved in these interactions. The pattern of herdmates involved in social interactions mirrored proximity in all cases, which was expected because limitations of video recording dictated that social interaction required a proximity of 2 body lengths. However, being proximate did not imply active social interaction; and, therefore, the number of samples proximate to a given herdmate was greater than or equal to the number of samples having social interaction with that herdmate, resulting in unequal percentage of time between proximity and social interaction in each habitat. Additionally, the adult females and M-juvenile spent between 2% and 9% of their time proximate to and interacting with adult bulls in the previous habitat, and only one bull was re-integrated with the herd in the new habitat and only for 6 months.

Female 1 (Figure 8a) was proximate to and interacted with all of her herdmates more evenly in the previous habitat, with only an 11% difference across herdmates in proportion of social interactions compared to 25% across herdmates in the new habitat. In the new habitat, her time spent interacting with Female 2 and M-juvenile decreased significantly (Female 2: W = 39,389, *p* = 0.049; M-juvenile: W = 38,737, *p* = 0.025), and she spent a larger portion of her time interacting with Female 3 and F-calf. Interestingly, the herdmates with whom Female 2 (Figure 8b) spent the most time proximate to and interacting with in the new habitat were opposite to those in the previous habitat. In the previous habitat, she spent the largest portion of her time proximate to and interacting with Female 1 and M-juvenile, whereas in the new habitat she spent the largest portion proximate to and interacting with Female 3 and F-calf, with significant increases in social interaction with Female 3 (W = 45,015, *p* = 0.005) and F-calf (W = 46,562, *p* < 0.001). Female 3 (Figure 8c) spent the majority of her time with her calf in both habitats. Her time spent proximate to and interacting with the other adult females increased in the new habitat (Female 1 interaction: W = 45,211, *p* = 0.006; Female 2 proximity: W = 46,024, *p* = 0.002), while her interaction decreased with M-juvenile (W = 38,601, *p* < 0.001).

F-calf (Figure 8d) spent the majority of her time with her mother in both habitats, but spent less time proximate with her mother (W = 22,863, *p* < 0.001) and more time with the other adult females in the new habitat, with an increase in social interaction involving other adult females (Female 1: W = 38,371, *p* = 0.011; Female 2: W = 40,482, *p* < 0.001) combined from 6% of her interactions in the previous habitat to 35% in the new habitat. She also showed a decrease in social interaction with M-juvenile (W = 33,509, *p* < 0.001) from 18% down to 4% of her interactions.

M-juvenile (Figure 8e) showed a decrease in proximity and social interactions with Female 3 (W = 35,162, *p* = 0.002), and an increase with F-calf (W = 34,401, *p* < 0.001). Although M-juvenile showed an increase in interactions with F-calf, she showed a decrease with M-juvenile. This inconsistency in herdmate proximity and social interaction for M-juvenile and F-calf between when he was the focal individual versus when she was the focal may be due to temporary separations of M-juvenile from Female 3 and F-calf surrounding estrus.

**Figure 9:**
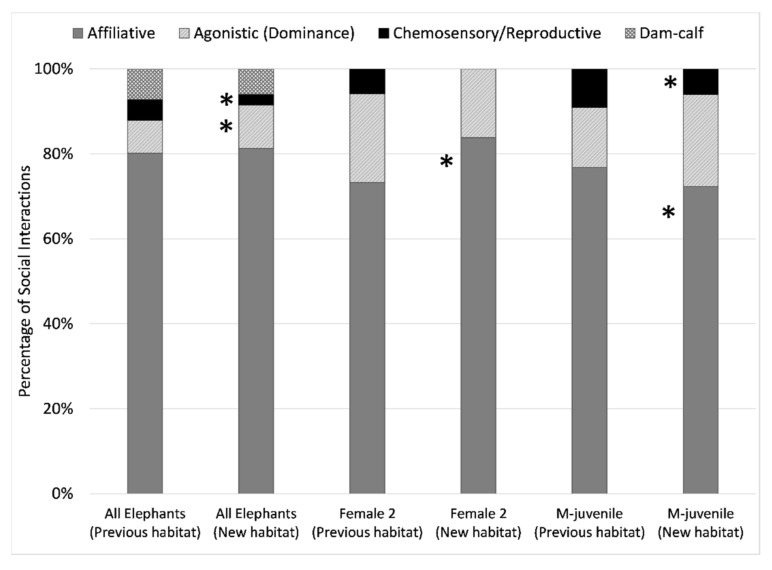
Percentage of functional types of social behaviors for all elephants combined and for individuals exhibiting significant differences between habitats. Asterisk (*) next to the bars indicates statistically significant difference between habitats when comparing a functional type of social behavior.

In comparing social function of behaviors, affiliative behaviors comprised the majority of all social interactions across all individuals, ranging from 72% to 91%. There was no significant difference in affiliative behaviors between habitats for all elephants combined (Figure 9), but all individuals exhibited a significant change in social partners for affiliative interactions that mirrored the changes for all social interactions combined (Figure 8). Behaviors indicating arousal were observed only in the previous habitat and were rare (less than 1%).

Agonistic or dominant/submissive behaviors increased (W = 1,014,842, *p* = 0.045) in the new habitat for all elephants combined from 8% to 11% of interactions. On an individual basis, only Female 1, Female 2, and M-juvenile exhibited a sufficient proportion of agonistic behaviors for analysis. The habitat differences were not significant in the proportion of interactions that were agonistic, the social partners in those interactions, or the role of the individual as the sender (instigator) or receiver (recipient). However, changes in social partners and social role in these interactions are noteworthy as they reflect changes in herd dynamics, and any lack of significance could be due to the relatively low occurrence of these behaviors.

Female 2 (Figure 9) experienced an increased (W = 45,247, *p* = 0.049) proportion of affiliative interactions in the new habitat (from 73% up to 84%). Female 2 was usually the receiver in agonistic interactions, but this decreased (from 78% down to 72%) in the new habitat. In the previous habitat, Female 2 engaged in agonistic interactions with M-juvenile (59%), Female 1 (29%), and Female 3 (12%), but not F-calf. In the new habitat, her agonistic interactions decreased with M-juvenile (down to 28%) and Female 1 (down to 11%), and increased with Female 3 (up to 28%) and F-calf (up to 33%). Female 1 was usually the receiver in agonistic interactions in the previous habitat (63%), but the instigator in the new habitat (64%). In the previous habitat, Female 1 engaged in agonistic interactions most frequently with M-juvenile (38%) and F-calf (25%). In the new habitat, her agonistic interactions decreased with M-juvenile (down to 20%), increased with F-calf who she engaged most with (up to 40%), and showed no notable change with Female 2 and Female 3.

M-juvenile (Figure 9) experienced a decreased (W = 31,714, *p* < 0.001) proportion of affiliative behaviors in the new habitat (from 92% down to 86%) and a decreased proportion (W = 36,308, *p* = 0.030) of chemosensory or reproductive behaviors (from 9% down to 6%). M-juvenile was usually the instigator in agonistic interactions, and this increased (from 65% up to 76%) in the new habitat. In the previous habitat, M-juvenile engaged in agonistic interactions most frequently with Female 2 (69%), Female 3 (19%), and Female 1 (9%). In the new habitat, his agonistic interactions decreased with Female 2 (down to 29%), increased with Female 1 (up to 35%) and F-calf (up to 18%), and showed no change with Female 3.

#### 3.4.4. Behavior Changes during Construction Prior to Habitat Changes

There were no significant differences associated with construction alone in the previous habitat in time spent performing all active behaviors (social behaviors, interaction with food-delivery and non-food enrichment objects and habitat features, feeding/drinking, and locomotion) for individuals or all elephants combined.

Female 1 showed an increase (W = 15,090, *p* < 0.001) in habitat feature interaction during construction in the previous habitat (from 3% up to 11%), and a decrease (W = 12,462, *p* = 0.014) in repetitive behaviors (from 8% down to 3%). Female 2 showed an increase (W = 15,450, *p* = 0.011) in locomotion during construction in the previous habitat (from 13% up to 18%). Female 3 showed no significant changes in behavior during construction in the previous habitat. F-calf showed an increase (W = 8497, *p* = 0.007) in feeding/drinking during construction (from 9% up to 22%) and a decrease (W = 6343, *p* = 0.014) in habitat feature interaction (from 16% down to 10%). M-juvenile showed a decrease (W = 11,120, *p* = 0.043) in feeding/drinking during construction (from 50% down to 41%,) and an increase (W = 14,500, *p* = 0.012) in social interaction (from 13% up to 24%). His proportion of affiliative behaviors increased (W = 14,693, *p* = 0.003) during construction (from 63% up to 81%,), and he showed increased affiliative interactions with Female 1 (W = 13,970, *p* = 0.008) and F-calf (W = 13,977, *p* = 0.003), decreased affiliative interactions with his mother, Female 3 (W = 11,801, *p* = 0.025), and no difference with Female 2.

### 3.5. Resource Use

**Figure 10:**
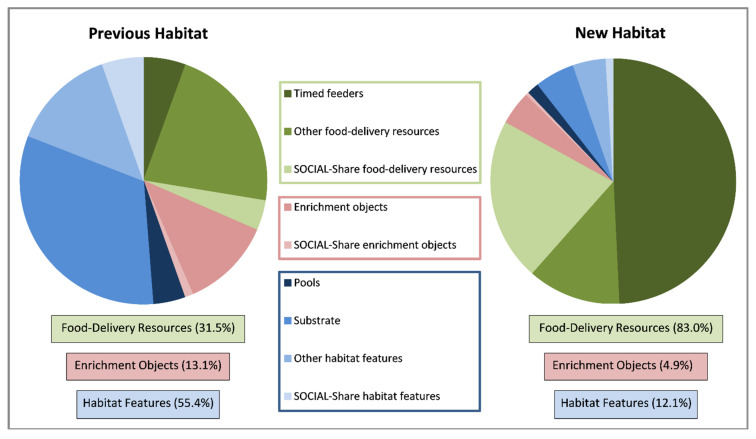
Interactions with resources in the previous and new habitat.

The percentage of time allocated to interacting with the diversity of resources available for elephants relative to all interactions with food-delivery resources, habitat features, and enrichment objects is shown in Figure 10. Interactions with food-delivery resources increased from 31.5% in the previous habitat to 83.0% in the new habitat. Behavior measures showed a decrease in interactions with permanent habitat features, and use of these habitat features relative to other resources decreased from 54.0% in the previous habitat to 12.1% in the new habit. Behavior measures showed no significant change in the percentage of time spent interacting with enrichment objects, but with the switch to feeding primarily from food-delivery resources, the use of enrichment objects relative to other resources decreased from 13.1% in the previous habitat to 4.9% in the new habit.

The proportion of time spent seeking food and feeding was higher (34% higher) than it was for interacting with enrichment objects and habitat features in the new habitat; therefore, the engagement with enrichment objects and habitat features was observed less frequently in the new habitat. It is important to note with our sampling methods of recording behavior 1 day per week with eight 2-min recording sessions, both the interaction behaviors and overall resource usage may have been underestimated even though the sampling interval of 30-s captured more rare behaviors in methods testing. It is also possible that the moving of objects to obtain food (e.g., moving a log under a hanging feeder) was underestimated given the hierarchy of the behavior ethogram. Elephants were observed engaging with the inside “rainfall” of the deluge system and the outdoor water cannon; however, these habitat features were not evaluated because they required caretaker interaction.

Interacting with all of these resources in a social context (i.e., sharing the resource) comprised 22.9% of the habitat resource interactions in the new habitat compared to 10.5% in the previous habitat. Sharing of food from, or sharing space around, a food-delivery resource was the primary driver for the increase, comprising 25.9% of all food-object interactions in the new habitat compared to 12.5% in the previous habitat. Simultaneously, interacting with the same enrichment object comprised 8.6% of all enrichment-object interactions, similar to the previous habitat (8.5%), and using the same habitat feature in a social context (e.g., playing in the pool together) comprised 8.0% of all habitat feature interactions compared to 9.8% in the previous habitat.

## 4. Discussion

Results of this study illustrate that the welfare-based design of the new habitat achieved its desired goals. The elephants were more active, walking farther, and foraging and exploring more; they expressed a diverse range of species-typical behaviors with evolving herd dynamics across life stages; they also exhibited more autonomy in choosing different aspects of their environment with regular indoor/outdoor access and extensive resource use. Elephants experienced biologically meaningful challenges throughout the construction period and subsequent acclimation to a novel environment, and they responded in a healthy and adaptive manner. Utilization of the entire habitat without avoiding or being excluded from areas suggests more choice in whether to spend time together or move away to engage in other activities.

### 4.1. Walking Distance

With an average of around 16 km per day and a maximum of over 23 km, the elephants at the Oregon Zoo appear to walk at least as far as, and perhaps at times even farther than, their wild counterparts on a daily basis. Walking distances comparable to those of wild elephants have also been found in other studies of zoo elephants [37,38,93,94]. Movement patterns of free-ranging elephants have been measured under different environmental conditions using a variety of techniques, from radio and GPS collaring to observers following individuals and herds (reviewed in [38]). Reported daily, walking distances of wild African elephants are generally greater than for Asian elephants. Wild African elephants are reported to walk 3 to 12 km per day under normal environmental conditions [39,40,41,95], and can range up to 27 km per day or more for landscape-level movements such as seasonal migrations or dispersal [96] or under extreme conditions such as in water scarce regions of Namibia [31,97,98]. By contrast, wild Asian elephants in Myanmar were shown to walk from 1.3 to 7.3 km per day [99]; in Sri Lanka, shorter daily walking distances were found in elephant groups with nursing calves (1 to 3.5 km) or juveniles (3 to 8.5 km) [62], as cited in [38]. Another measurement of movement is distance displaced, which measures a straight-line change in location as a result of walking any pattern. By measuring displacement, Alfred et al. [100] found that Bornean elephant herds travelled an average of less than 2 km per day with a maximum of 4 km per day. Perhaps the longer distances measured in zoo elephants is in part due to the fact that they are secure to move as individuals and their movement is not limited by group composition or group needs. Free-ranging herds with a nursing calf or old or sick elephants will have to move more slowly; however, elephants in a zoo can move as individuals in safety. Additionally, it is important to note that the methodology for measuring movement in free-ranging animals is often based on relatively low sampling rates to preserve battery power or because of observer limitations, which likely results in an underestimate of total distance walked.

Food and water availability have been shown to influence walking distance in free-ranging Asian elephants [99,100], and thus one of the challenges in a zoo setting is to encourage walking given ample food provisions. Holdgate et al. [37] found individual differences in walking distances associated with demographic, social, housing, and feeding-related variables. More diverse feeding regimens correlated with increased distances, and elephants fed on an unpredictable schedule walked 1.29 km/day further per day than elephants fed on a predictable schedule. One explanation given was that dynamic feeding regimens lead to increased expression of exploratory behavior, which was also seen in the current study with elephants checking food-delivery resources while moving through the habitat. These findings associating increased walking with feeding regimens support the assessment by Veasey [101] that foraging is of psychological significance to Asian elephants, and that walking may be a largely appetitive behavior synonymous with foraging—that walking in Asian elephants is not a behavior expressed for its own sake, but rather as a means to secure nutritional, social, or physical resources, and as such, walking is largely appetitive.

Finally, improvements in muscle tone and foot health observed by animal care staff indicated improved overall health in the new habitat, deemed as a consequence of increased activity levels, varied body movement to obtain food, movement through a more varied topography, and walking on natural substrate. Although a previous epidemiological study found that walking distance was not correlated with either foot or musculoskeletal health [37], for the elephants in our study, walking on natural substrate in the new habitat and getting more daily exercise are deemed to be factors important to improving body condition and foot health [12,13,48].

### 4.2. Reproductive and Adrenal Hormones

The continued and regular ovarian cycling observed in females throughout the construction period and transition to the new habitat represents one indicator of normal reproductive health and welfare for the female elephants in this study. In our previous long-term study of gonadal [75] and adrenal activity [102] of female Asian elephants, we found that major life events—births, deaths, changes in herd structure—had minimal effect on estrous-cycle dynamics over time and adrenal responses were activated only short-term, suggesting that the Oregon Zoo’s female elephants are quite resilient and maintain normal reproductive and adrenal health. Historically, male elephants at the Oregon Zoo exhibited typical annual musth cycles [71]. M-juvenile exhibited his first signs of musth associated with elevated testosterone at 6 years of age, during the construction phase. In wild Asian elephants, musth has been observed as young as 14 years of age [103], and age 15 is generally considered adult [104]. Studies have shown that elephants in captivity can reach sexual maturity at an earlier age than their wild counterparts, perhaps as an artefact of nutrition [105] and social factors [105,106]. Musth onset in Asian elephants in captivity has been reported as young as age 7 [107] using behavioral and visible indicators of musth. Brown et al. [70] included endocrine measures to determine age of first musth at age 12 and 15 in Asian bulls. So, it appears that M-juvenile experienced his first musth at a relatively young age. Further research is needed on factors influencing musth onset in zoo-housed elephant bulls.

Higher variability in adrenal activity during the construction phase suggests adaptive and normal adrenal responses to life challenges, changes, and excitement. The decline in median FGM concentrations and variability in the new habitat suggests that the elephants acclimated well to their new environment, with no evidence of a chronically elevated adrenal response. Furthermore, the elephants were observed closely by caretakers during introductions to any new spaces, and all elephants appeared to transition smoothly and adapt well to changes throughout this period. This might be attributed in part to the protective effects of ‘social’ support from conspecifics [108,109] and from caretakers [110,111,112,113] given the strong bonds between these elephants and also with their caretakers. Plotnik and deWaal [109] observed increased affiliative responses of elephants towards individuals exhibiting distress, suggesting the capacity of elephants for reassurance, and bouts of affiliative behavior towards aroused individuals have also been observed among this group of elephants. Affiliative interactions among conspecifics has been shown in some animals to buffer the stress response [108].

The construction phase included not only disturbances caused by heavy construction and associated noise, but also elements of unpredictability with introductions to new habitat areas and significant changes to formerly familiar areas. Studies have shown that unpredictable environmental conditions can result in elevated concentrations of glucocorticoids in vertebrates [114]. Jakob-Hoff et al. [115] observed increased vigilance and apprehension in zoo elephants exposed to short periods of construction noise, but construction disturbance occurred over an extended time in the current study, so there was potential for habituation and there was no evidence of any lasting negative effects. Although the females in this study historically have not exhibited signs of chronic stress [102], Carlstead and Brown [116] argue that high variability in longitudinal FGM data may be a better measure of increased reactivity than increased mean levels, because the best indicator of chronic stress in animals subjected to intense stressors of long duration or repetitiveness may be more frequent or exaggerated glucocorticoid responses to new and acutely presented stressors (Mason et al., in prep).

Individual variation in adrenal hormones in response to exogenous stimuli is ubiquitous in all vertebrate groups [117], and was also observed in this study, with a wide range in FGM concentration medians and variability across elephants. Female 1 had the largest increase in interquartile range (IQR) of FGM concentration during the construction phase, suggesting a stronger adrenal response compared to the other elephants. She was also the most tentative in exploring new areas and took longer to learn the new feeding strategy. Her temperament and disposition may have been a contributing factor influencing her adrenal response, which has been shown in many species [118]. Although Female 2 did not show a return to previous median levels in the new habitat, declines in FGM concentrations immediately following introduction to new areas indicates normal adaptive responses. Her social interactions may have been a confounding factor in sustained concentrations in the new habitat. In this time period, Female 2 spent more time with M-juvenile than did the other females, and although her agonistic interactions with M-juvenile decreased in the new habitat, courtship and mating-related behaviors were observed by staff, and these interactions can temporarily increase adrenal activity and thus affect overall glucocorticoid concentrations [51] For Female 3, the confounding factor of reproductive state needs to be considered. Glaeser et al. [102] showed that serum cortisol covaried with reproductive state in female Asian elephants, and with her first calf, Female 3 exhibited her highest mean serum cortisol concentrations during lactational anestrous, followed by pregnancy, then normal cycling. In the current study, she was in lactational anestrous for the first 34 of 140 weeks of construction, and then resumed normal cycling while continuing to nurse her calf, so reproductive state alone does not account for her increase in median concentrations during construction. However, the metabolic demand of lactation [119] and the “stress” of early calf-rearing [120], albeit generally positive in nature, could explain increased median levels and variability in the timeframe of construction in this female.

F-calf showed higher median and greater variability in adrenal activity during the construction phase, a pattern similar to other elephants in this study. F-calf’s first four years of life were monitored during this study, and her development stages may have affected her adrenal activity as well, but it is not known whether or how the stress response changes with early development in elephants. Vijayakrishnan et al. [121] found that mean baseline FGM concentrations of free-ranging Asian elephant calves were generally lower than those of all other age classes, but calves exhibited the strongest response to a major stressor of being driven out of plantations. In contrast, F-calf exhibited intermediate levels and variability compared to other elephants in this group. In a model mammalian species, the rat, Sapolsky and Meaney [122] found that immature animals had a prolonged stress response compared to adults. Glucocorticoids were secreted in response to stressors in the late fetal period with concentrations within range of the adults, followed by a 2-week adrenocortical quiescence in the post-natal period, then as the capacity to initiate a stress response emerged with age, the termination of the stress response was delayed compared to adults. It is apparent that further investigations are needed to elucidate how the stress response changes with development in young elephants.

### 4.3. Behavior

Overall, the elephants exhibited a diverse range of species-typical behaviors and natural social dynamics of a healthy herd in both habitats. Activity budgets differed between the new and previous habitats, with increased activity levels, increased locomotion, increased foraging, and decreased repetitive behaviors in the new habitat, indicating that the more enriched and complex environment improved behavioral indicators of well-being. Interestingly, the elephants also exhibited different social choices between the two habitats, with changes in whom they primarily associated with and how they interacted with their herd mates.

In evaluating the behavioral repertoire of animals in human care, it is important to define “naturalness” in terms of similarity to their wild counterparts in order to identify desirable species-typical behaviors. Veasey et al. [123] defines natural or wild behavior as behavior expressed by an animal subject to environmental and evolutionary pressures with minimal human intervention. Yeates [124] broadens this definition stating that natural behavior means human intervention has had zero or negligible effect. Although this is generally not the case in managed populations, Yeates [124] argues that if a behavior occurring in the wild is also expressed in managed and captive settings, then the behavior is deemed natural. So, the relationship between naturalness and welfare deserves attention. The evolutionary adaptation responsible for ‘wild’ behavior is likely optimized by survival and reproduction rather than by welfare [124]. Furthermore, it is possible that the expressions of ‘wild’ behaviors correlate with enhanced welfare, rather than cause enhanced welfare, making the consequence of the behavior more important than expression of the behavior itself [123]. Veasey et al. [114] suggest that comparisons with wild animals should be used in combination with other indicators to demonstrate that the consequence of not performing ‘wild’ behaviors or performing them at a different rate results in compromised welfare, with the reasoning that expression of these behaviors in the wild does not eliminate the possibility that the animal is suffering. Behavior patterns may be altered in animals in human care with a different health status from that of their wild counterparts who face untreated disease, parasitism, or injury, and with the relaxation of selective pressures of food acquisition, predation, territorial aggression, etc., making a direct comparison misleading [8,101,125]. The behaviors measured in this study included behaviors expressed in the wild for a group of elephants of mixed ages and both sexes, with additional indicators to determine whether these behaviors are beneficial to each individual.

#### 4.3.1. Activity Levels and Locomotion

The increase in percentage of time spent engaging in active behaviors indicates that the new habitat indeed succeeded in the goal to increase overall activity levels for the elephant herd. Locomotion behavior increased for all individuals but lacked significance for some. However, our behavior data collection methods categorized ‘locomotion while feeding’ as a feeding behavior; therefore, walking and feeding at the same time, common foraging behavior in elephants, was not included in the locomotion behavior category. This likely resulted in an underestimate of locomotion monitored through behavior observation. For Female 1, the doubling of measured walking distance via GPS tracking, but the lack of a significant concomitant increase in locomotion measured through direct behavior observation, is therefore likely due to the different sampling methods and frequencies. Behavior observations were conducted only during the zoo’s public open hours, and were recorded once a week for 16 min with a sampling interval of 30 s, whereas GPS data were collected once every two weeks for 24 h with a sampling interval of 5 s. The GPS data are therefore more reliable representations for overall movement and walking distance.

#### 4.3.2. Foraging, Feeding, and Exploratory Behaviors

Allocation of time to daytime foraging and feeding was similar to Asian elephants at other zoological institutions [126,127]. In the previous habitat, care staff provided an array of simple puzzle feeders and scattered food to extend feeding time and encourage investigation during and after staffed hours. Our findings indicate that the food-delivery resources designed into the new habitat provided more diverse foraging opportunities and promoted natural browsing behavior of movement between dispersed resources, which was also supported by observed increases in locomotion and daily walking distances. Furthermore, sharing of food resources comprised a greater percentage of the activity budget and of all social interactions in the new habitat, resulting in increased foraging and feeding time in the new habitat.

The switch from food directly provided by caretakers towards independently seeking food available through various types of feeding devices dispersed throughout the new habitat appears to provide a superior foraging and feeding experience for the elephants. Ultimately the ability to express more natural and diverse foraging behaviors may in turn help meet the psychological needs of this species. Foraging and feeding are intrinsically linked, but functionally they are different. Foraging represents the appetitive phase of acquiring nutrients which, for wild elephants involves social interaction, information gathering and processing, group and individual decision making, and locomotion, whereas feeding is the consumption phase and involves less cognition but has more direct survival impact [101]. Both appetitive and consummatory behaviors are important to the fulfilment of an animal’s motivational needs, but it has been suggested that appetitive foraging behavior more than consumption increases aspects of “perceived control” over the environment (reviewed in [128]). In an assessment of psychological priorities for captive Asian elephant welfare, Veasey [101] demonstrates the importance of providing species-appropriate feeding, social, and cognitive opportunities, with the greatest priority being appetitive behaviors essential for survival in the wild, and therefore recommends that wherever possible, animals should be empowered to satisfy their own needs by expressing appropriate motivated behaviors and cognitive processes, rather than having these needs met through direct provisioning by human caretakers. As part of their new foraging experiences, elephants in this study were able to use cognitive processes to learn a new feeding regimen. Furthermore, finding food on a daily basis requires motivated behaviors of walking, exploration and manipulation, climbing and reaching, as well as social interaction and decision making.

#### 4.3.3. Repetitive Behaviors

Repetitive behaviors in the new habitat were exhibited in only two females and occurred at low rates (average 4%), much lower than those reported for the AZA Asian elephant zoo population (15.5–24.8% [10]). In the new habitat, a decrease in frequency was observed in one female, and a change in bout pattern was observed in the other female, which appeared to be primarily related to anticipation of routine feeding as observed in other studies (e.g., elephants [127], polar bears [129], stabled horses [130], carnivores [131]). This apparent feeding anticipation subsided as this individual learned to seek food independently, so it is arguable that this anticipatory behavior served a function. The ability of animals to anticipate the arrival of predictable events is hypothesized to play an important role in stimulating appetitive searching behaviors [35]. However, when food is presented in a predictable manner, these appetitive behaviors can become disassociated with their original function and develop into stereotypic behaviors [35], which are repetitive and invariant in form, and with no apparent function [132,133]. Repetitive behaviors have been well-documented in elephants living in human care (reviewed in [10]), but their lack of function and inference of current or compromised welfare state have been debated [133,134]. In some species, food-associated anticipatory stereotypies have been disrupted and reduced by providing animals with temporally and spatially unpredictable feeding [47]. The observed decreases in repetitive behavior in the new habitat may be attributed to increased opportunities for expressing species-specific foraging and feeding behavior [29,47,127,135,136,137] and to other aspects of a more enriched environment [26,27,138,139], indoor/outdoor access most of the year [10,140], and perhaps also to time spent with juveniles [10], which has been shown to stimulate play or teaching behavior in older herdmates [18].

#### 4.3.4. Social Behaviors and Partners

Elephants made different choices between the two habitats in whom to spend time close to and interact with, which reflected natural and evolving herd dynamics with a young male and female calf transitioning through life stages. Young elephants move further from their nearest neighbors with increasing age to explore their environment [17,141,142], which was observed in both M-juvenile and F-calf. Although some changes in herd dynamics were related to temporal factors of sexual and social development, the new habitat provided social opportunities to better support and allow for these evolving dynamics. M-juvenile’s decrease in social interactions reflected his changing role in the herd and the gradual process of becoming more independent from his natal group [19,143,144]. M-juvenile was still a juvenile in the previous habitat, and after exhibiting his first musth during the construction phase, his social interactions with the females became less affiliative and more dominant at least during musth. Furthermore, as an adolescent in the new habitat with evidence of sexual maturity, management necessitated temporary separation from related females during the receptive part of their female reproductive cycle to avoid undesirable pregnancies (i.e., inbreeding).

F-calf’s changes in proximity and social interaction and her mother’s decrease in protective behaviors in the new habitat also reflected natural development changes. In wild elephants, once nursing infants are no-longer milk-dependent, they spend less time in close proximity with their mother [141], and the distance between calves and mothers increases gradually with age [17]. However, juvenile elephants still spend the highest proportion of their time with their mothers and other adult females [14,142,143], which was also observed in F-calf. Jayantha et al. [142] found that juvenile Asian elephants spent about half their time near their mothers and other adult females, and the majority of remaining time (37%) near other juveniles, with social interactions observed to be most frequent with other juveniles, followed by infants. Time spent near sub-adults of either sex was less than 10%, and time spent near adult males was negligible. F-calf also spent the majority of her time near her mother and other adult females in both habitats, but her time close to and engaging with M-juvenile decreased in the new habitat as he was maturing into an adolescent. The relative time F-calf spent with each herd mate appeared to be similar to what would be expected in herds with one or two young elephants.

Not surprisingly, affiliative behaviors comprised the vast majority of social interactions in both habitats. Among adult females, there is evidence the rate of affiliative and aggressive interactions is influenced by estrous cycle phase [145]; however, given that these females have exhibited normal regular reproductive cycling over their lifetime [75] and during this study, any changes in these types of interactions could be attributed to other factors. The observed agonistic behaviors are important as they serve to establish and reinforce social dominance status, which is essential for maintaining social harmony in captivity [146] and avoiding escalation to aggression. Social dominance in elephants is achieved by a combination of size, age, temperament, and experience [43,143,146,147,148], although dominance hierarchies in wild Asian elephant populations in productive habitats have been shown to be somewhat weaker and nonlinear when compared to African elephants [149]. M-juvenile was usually the instigator in agonistic interactions, which increased in the new habitat. In the previous habitat, these interactions were primarily with the adult females and mostly with Female 2, whereas in the new habitat, the proportion of agonistic interactions with each herdmate was more uniformly distributed and included F-calf, which provides further evidence of M-juvenile’s changing role in the herd. In turn, Female 2’s increase in affiliative behaviors and decrease in agonistic interactions with M-juvenile and Female 1 in the new habitat suggest a more equitable social environment for this individual. Furthermore, the majority of Female 2’s agonistic interactions in the new habitat were with F-calf, possibly to establish social dominance as F-calf had grown to almost the same size. Like Female 2, the majority of agonistic interactions in the new habitat for Female 1 were with F-calf, likely to discipline or assert dominance.

The presence of adult bulls influences female behavior, which in the previous habitat accounted for courtship and arousal behaviors not observed in the new habitat, and higher proportions of chemosensory and reproductive behaviors. Pinto et al. [150] found that a group of captive female elephants in the presence of an adult bull exhibited stereotypic behaviors less frequently, spent less time foraging, and more time standing and engaged in courtship behaviors compared to when the bull was absent. However, the percentage of time interacting with the bulls in the previous habitat did not account for the lower proportion of time spent foraging and the higher proportion of time stationary.

Percentages of time spent in proximity to herdmates reflected that of other socially bonded elephants [151]. Overall decreased proximity among herd members in the new habitat combined with sustained, or increased proportions of time actively engaged in social behaviors implies increased choice of whether to stay near or move away from a herdmate to engage in a different activity when not actively socializing. Although providing more opportunities for individual choices was a priority in the design of the new habitat, and perhaps decreased proximity was a consequence, elephant care staff also created opportunities for group bonding with large browse feedings that encourage the herd to eat together.

#### 4.3.5. Behavioral Changes Associated with Construction

In assessing the impact of construction activities while in the previous habitat, the consistency in activity level and the adaptive and appropriate changes in certain types of behavior (e.g., increases in exploratory behavior) suggest that these elephants showed no negative response to the construction alone, congruent with the adrenal hormone results. This group of elephants showed resilience through adaptive behavioral and adrenal responses to disturbance and increased environmental complexity. The reduction in stereotypy and increase in habitat interaction exhibited by Female 1 coincident with construction could be due in part to her ever-increasing time spent with a young calf. Although F-calf nursed throughout the entire study, her increase in feeding/drinking during construction was attributed to her development and transition to solid foods. Her decrease in habitat interaction was also observed in the new habitat compared to the previous habitat, and any changes in exploratory behavior could also be attributed to calf development. It is plausible that the increased affiliative interactions of M-juvenile with Female 1 could be related to social support during a period of disturbance; however, the increase in social interaction with F-calf and decrease with his mother was the same pattern observed in the new habitat, so it is more likely that these changes were related to typical herd dynamics with a developing young male.

#### 4.3.6. Space and Resource Use

The layout of spaces in the new habitat gives bulls the opportunity to join and leave the herd, and for females to stay together as a group or spend time alone, choosing their social partners throughout the day. Elephants also have increased choice in environmental conditions with indoor/outdoor access throughout the year, mist/rain stations, and varying substrates. GPS data showed that some individuals chose to leave heated indoor areas and spend the majority of their time outdoors on colder days, while others chose to remain indoors, and that the elephants utilized the entire area of the new habitat with no evidence of avoidance of or exclusion from particular spaces.

Our findings on resource use highlight the value of varied types of food distribution, with timed feeders and overhead feeders being instrumental in encouraging foraging throughout the habitat. Among the food distribution resources in the new habitat, all were used extensively with exception of the cement herd feeders, which provided another climbing opportunity and required reaching down to get food. In the timeframe of this study, the feeding holes big enough for adult trunks were also big enough for the calf’s foot, so holes were plugged and food was not put inside to encourage climbing. Despite the relative decrease in interactions with permanent features in the habitat (e.g., scratching surfaces, large logs), the elephants manipulated their environment more by pulling, pushing, kneeling, stretching, etc. to obtain food items or to move objects for other reasons (including play behavior). It was not uncommon for M-juvenile or the adult females to move a log or stump under an overhead feeder then stand on it to reach food more easily. The larger and deeper pool also allows for more water-based activity and exercise for all elephants, and the elephants clearly enjoyed swimming and resting in the pool.

It is important to note that approximately one quarter of the resource interactions in the new habitat were in a social context involving sharing of resources, with the majority of sharing being related to food. Although dominance/submissive interactions were observed, including displacement from resources, there was no evidence of differential space use over time in relation to age or dominance in this group of elephants, contrary to findings in some studies of captive elephants [152] and other herd species [153]. This equity in resource use can be attributed in part to food distribution strategies, but also to social dynamics being primarily affiliative in nature.

### 4.4. Environmental Complexity and Provisions for Choice and Control

While the new habitat was more complex and the dynamic feeding regimen introduced unpredictability which encouraged foraging, another program goal was to offer the elephants greater control or greater self-determination. Environmental complexity and provisions for choice and control in daily life have been shown to influence the welfare of animals in human care in zoo, agricultural, and laboratory settings [21,22,24,25,36,154,155,156,157,158,159,160,161]. Carlstead and Shepherdson [158] emphasize the importance of complexity in developing animals, who learn that performing active behavior produces appropriate functional outcomes, which influences their ability to adapt to novel situations. By providing captive animals with more complexity, in physical, temporal, social, food, cognitive, or sensory domains, we are also providing them more choices, and vice versa [25,154,158]. The links between complexity, choice, and control are complicated, particularly those related to control, and the concept of control must first be understood. Carlstead and Shepherdson [158] and Sambrook and Buchanan-Smith [24] offer similar views of “control” as a psychological construct in which the animal has developed contingencies between performance of a behavior and its functional outcome, with a direct relationship between the animal’s behavior and its consequences. Therefore, a particular event is controllable if the likelihood of the event occurring depends on the behavior of the animal. Human studies have shown that “perceived control” has benefits to well-being even in the absence of actual control [162,163,164,165], and Kagan and Veasey [155] differentiate perceived control in animals as awareness of the choices and opportunities it has in its environment. Finally, the opportunity for choice enhances an individual’s perception of control [22,165]. Simply stated, complexity affords more opportunities for choice, and more choice allows a greater degree or perception of control.

One major difference between the environments of captive and wild animals is the reduced complexity leading to increased predictability, and the reduced amount of environmental control animals have in daily life [166], which ultimately restricts opportunities to exercise cognitive skills to solve problems and make decisions [21]. Control and predictability are inextricably linked: if an animal can control something, he or she can predict it will happen [25]. Observed choices in sociality and environment could arguably show greater control because the elephants’ behavior affected the outcome, but the balance of predictability and control in food acquisition needs further discussion. As a means of survival, animals in a less predictable environment experience higher appetitive motivation for exploration and foraging (reviewed in [35]). The new habitat had three types of predictability (or unpredictability) related to food acquisition—temporal predictability, spatial predictability, and signaled predictability. Food was less predictably distributed in time and space in the new habitat, but elephants did learn that if they walked around and checked food sources regularly, they would find food eventually, and by doing so they gained more control and provided more predictability themselves. A reliable signal was the audible sound of a timed feeder tray dropping that could be heard if an elephant was close enough, and a signal of varied reliability was the visual signal of another elephant approaching or at a feeding resource.

The elephants used cognitive skills in learning new feeding strategies and exploring new areas. Sambrook and Buchanan-Smith [24] speculate that the acquisition of control may be a more enriching process than the execution of control, and this acquisition requires cognitive skills. The process of learning itself and the provisioning of learning opportunities (or cognitive challenges) to zoo animals may offer welfare benefits beyond immediate survival, including improved psychological well-being and enhanced cognitive function [167]. Foraging in a new area and in new ways involves cognitive mechanisms, such as environmental perception, memory, and problem solving. Animals may also experience a positive emotional reaction to learning opportunities that are sufficiently challenging but within their skill set [21]. The challenge to management then becomes that of maintaining opportunities for choice and for cognitive challenge. In this case, the challenge is to maintain the cognitive component in the feeding strategy, in addition to the motor activity (e.g., walking, reaching, manipulation) and extension of feeding time (e.g., timed food disbursement across 14–16 h per day).

The compression of space available to wide-ranging animals (e.g., large carnivores, elephants, primates) in zoos is often perceived as detrimental to their welfare; however, there is evidence of effective strategies in safeguarding welfare by addressing habitat quality in a taxa-specific manner, providing appropriate mental stimulation and cognitive opportunities [33]. The lack of association in previous studies of habitat size to walking distance [37] or activity level [168] challenges the assumption that elephants will choose to walk greater distances if provided with more room. Furthermore, the aforementioned associations of increased walking distance and activity level with dynamic feeding regimes and habitat complexity suggest that space may be important to the extent that it can support greater habitat flexibility and complexity to promote species-typical behaviors and allow individuals more choices [33,159]. In the current study, habitat size was also increased compared to the previous habitat, so the relationship between size and walking distance, or activity level, could not be directly tested due to the multitude of other factors involved.

An environment that provides sufficient complexity, choice, and control will allow animals to thrive within their own capacity, and to develop abilities to cope with the challenges they may face [32]. Mellor [169] suggests that environments which provide opportunities for animals to engage in behaviors they find rewarding (e.g., exploration, food acquisition, social bonding, mating, care of young) can generate various forms of comfort, pleasure, interest, confidence, and a sense of control.

## 5. Conclusions

This study provides data on how environmental factors influence welfare outcomes in an endangered species, with the ultimate aim of using evidence-based management to provide animals with an environment in which they can thrive and experience optimum welfare. A new habitat for a group of zoo-housed Asian elephants constituted major environmental changes, with novel habitat areas and features, and a new feeding regimen. Findings from this study indicate that increased habitat complexity, improved spatial features, indoor/outdoor access with choice in environmental conditions, and natural substrates, as well as a multitude of areas to walk to and explore, all helped to create a more beneficial environment as a foundation for improved welfare. The use of integrated food-delivery technology (e.g., timed feeders, mechanisms for overhead feeding, etc.) functioning in a semi-autonomous manner supported species-typical foraging and feeding patterns, and afforded a more dynamic feeding regimen with less predictability in time and space, better satisfying the elephants’ appetitive motivations important for psychological well-being.

Promoting good welfare should focus on the individual’s perspective, and take into consideration how welfare is influenced over time by life stage, reproductive state, environmental factors, and social dynamics. With a focus on meeting the physical, physiological, psychological, and social needs of elephants in human care 24-h a day and through all life stages, new habitats should be designed with sufficient complexity to provide opportunities for choice, thus allowing a greater degree or perception of control. Ultimately, habitat design should afford flexibility in management, with appropriate space and resource distribution to support evolving herd dynamics and social equity for individuals across their entire lifespan.

## Figures and Tables

**Table 1:** Life history summary of the Asian elephants evaluated in the study at Oregon Zoo (OZ).

Elephant	Sex ♂/♀	Origin	Date of Birth or Transfer to OZ	Age at Start of Sample Collection
Female 1	♀	Zoo-born Oregon Zoo	26 December 1982—Birth	29 years
Female 2 ^1^	♀	Wild Borneo, Malaysia	~1993—Birth 20 November 1999—Transfer to Oregon Zoo	19 years
Female 3	♀	Zoo-born Oregon Zoo	31 August 1994—Birth	18 years
M-juvenile ^2^	♂	Zoo-born Oregon Zoo	23 August 2008—Birth	4 years
F-calf ^2^	♀	Zoo-born Oregon Zoo	30 November 2012—Birth	2 Months

^1^ E. m. borneensis, ^2^ Mother is Female 3.

**Table 2:** Elephant Behavior Ethogram (abbreviated version).

**Proximity**	**Definition**
Proximate	Focal animal is within 2 body lengths of or in contact with another individual (defined as 2 adult female body lengths, c.a. 10 m.)
Proximity Not Visible	Focal animal or other elephants are not visible enough to determine proximity.
**Behavior Category**	**Definition**
Behavior Not Visible	Elephant or activity is not visible enough to determine the behavior.
Social Interaction	Interacting with another elephant in a social context, either with physical contact (e.g., trunk twine, play) or without physical contact but within 2 body lengths (e.g., sharing food, displace).
Food-Delivery Object Interaction	Interacting with any object that distributes food, either permanent or provided by caretakers.
Feeding/Drinking	Acquiring/gathering and consuming (putting in the mouth) any food item without interacting with a food-delivery object. Drinking water, not bathing.
Enrichment (non-food) Object Interaction	Interacting objects that do not provision food and are not permanent (e.g., firehose ball or braid).
Habitat Feature Interaction	Interacting with features that are permanent in the habitat (e.g., dusting, bathing, digging, rubbing body, climbing on logs, investigating features with feet or mouth).
Repetitive Behaviors (Stereotypy)	Motor: Repeatedly performing a behavior for 3 or more consecutive repetitions without interruption (e.g., route tracing). Non motor stereotypy: Performing the behavior for 5 s or more.
Locomotion	Walking or running (fast walking) more than 2 body lengths in any direction, without stopping for 3 s or more.
Stationary	Any stationary state without engaging in another behavior for 3 s or longer. Standing, walking or shuffling (without moving 2 body lengths), sitting, kneeling, lying (prone or sternal).

**Table 3:** FGM concentrations (ng/g) in each habitat phase. Individual elephant, number of samples measured for FGM, and concentration median, interquartile range (IQR), and range (minimum–maximum).

Elephant	Previous Habitat				Construction Phase				New Habitat			
	N	Median (Range)	Q1–Q3	IQR	N	Median (Range)	Q1–Q3	IQR	N	Median (Range)	Q1–Q3	IQR
Female 1	19	124.21 (99.51–237.21)	112.71–138.57	25.86	85	162.81 (32.08–829.08)	120.34–260.41	140.07	49	116.57 (36.80–628.53)	98.22–158.55	60.03
Female 2	16	64.02 (43.37–81.65)	57.89–71.49	13.61	104	91.68 (21.60–356.66)	69.44–129.16	59.72	52	96.41 (29.59–162.84)	76.78–122.82	46.03
Female 3	12	141.91 (72.89–198.49)	124.80–168.32	43.52	97	193.59 (4.62–1355.22)	154.98–252.37	97.40	49	146.10 (14.20–231.54)	91.48–175.85	84.37
M-juvenile	18	65.57 (40.85–124.26)	57.44–71.20	13.76	115	103.50 (40.02–770.50)	81.55–147.03	65.48	53	79.79 (14.1–120.30)	63.92–91.13	27.21
F-calf	ND	ND	ND	ND	117	136.88 (7.12–730.38)	101.03–195.88	94.85	51	100.19 (26.05–262.32)	74.93–136.11	61.18

ND = no data.

**Table 4:** Comparisons of median FGM concentrations across and between habitat phases. Degrees of freedom (df) was 1 in all pair-wise comparisons.

Elephant	Kruskal-Wallis Test	Dunn’s Post-Hoc Pairwise Comparisons			
		Previous Habitat	Construction Phase	New Habitat	Dunn’s Multiple Comparisons
Female 1	χ^2^ = 16.04, df = 2, *p* < 0.001	X	X		Z = 1.90, *p* = 0.085
			X-higher	X	Z = 3.90, *p* < 0.001
		X		X	Z = −0.80, *p* = 0.425
Female 2	χ^2^ = 16.10, df = 2, *p* < 0.001	X	X-higher		Z = 3.93, *p* < 0.001
			X	X	Z = 0.06, *p* = 0.949
		X		X-higher	Z = 3.66, *p* < 0.001
Female 3	χ^2^ = 30.61, df = 2, *p* < 0.001	X	X-higher		Z = 2.81, *p* = 0.007
			X-higher	X	Z = 5.22, *p* < 0.001
		X		X	Z = −0.17, *p* = 0.87
M-juvenile	χ^2^ = 51.64, df =2, *p* < 0.001	X	X-higher		Z = 5.61, *p* < 0.001
			X-higher	X	Z = 5.55, *p* < 0.001
		X		X	Z = 1.83, *p* = 0.067
F-calf	χ^2^ = 51.81, df = 1, *p* < 0.001	ND	X-higher	X	

ND = no data.

**Table 5:** Comparisons of variability in FGM concentrations across and between habitat phases. Degrees of freedom (df) was 1 in all pair-wise comparisons.

Elephant	Levene’s Test for Equality of Variance				
	Across All Phases	Previous Habitat	Construction Phase	New Habitat	Dunn’s Multiple Comparisons
Female 1	F = 6.19, df = 2, *p* = 0.003	X	X-higher		F = 5.33, *p* = 0.023
			X-higher	X	F = 7.82, *p* = 0.006
		X		X	F = 0.72, *p* = 0.400
Female 2	F = 6.82, df = 2, *p* = 0.001	X	X-higher		F = 7.61, *p* = 0.007
			X-higher	X	F = 5.75, *p* = 0.018
		X		X-higher	F = 11.61, *p* = 0.001
Female 3	F = 2.89, df = 2, *p* = 0.059	X	X		F = 1.71, *p* = 0.194
			X-higher	X	F = 4.06, *p* = 0.046
		X		X	F = 2.83, *p* = 0.136
M-juvenile	F = 5.26, df = 2, *p* = 0.006	X	X		F = 3.24, *p* = 0.074
			X-higher	X	F = 7.36, *p* = 0.007
		X		X	F = 1.51, *p* = 0.224
F-calf	F = 8.09, df = 1, *p* = 0.005	ND	X-higher	X	

ND = no data.

## Data Availability

Data restrictions apply to the de-identified data underlying the findings of this study to protect the facilities and animals included in this study from potential abuse of this information. Data can be made available upon request to researchers who meet the criteria for access to confidential data, with approvals from registrars at the Oregon Zoo (Peter.Grimm@oregonzoo.org accessed on 30 June 2021).

## Appendix A. Elephant Behavior Ethogram (Full Version)

**Table A1:** LEVEL 1: Behavior categories.

**Proximity**	**Definition**
Proximate	Focal animal is within 2 body lengths of or in contact with another individual (defined as 2 adult female body lengths, c.a. 10 m)
Proximity Not Visible	Focal animal or other elephants are not visible enough to determine proximity.
**Behavior Category**	**Definition**
Behavior Not Visible	Elephant or activity is not visible enough to determine the behavior.
Social Interaction	Interacting with another elephant in a social context, either with physical contact (e.g., trunk twine, play) or without physical contact but within 2 body lengths (e.g., sharing food, displace). LEVEL 2: Social behavior
Food-Delivery Object Interaction	Interacting with any object that distributes food, either permanent or provided by caretakers. Behavior includes both seeking food and feeding: Investigating food-delivery object with trunk obvious attempts to reach and manipulate the food-delivery object (e.g., the object is in a position that is challenging to reach), manipulating and putting food item in the mouth before/after interacting with the object. Food Object vs. Enrichment objects: For items that can be either (e.g., barrel), determined by whether the elephant appears to be eating before/after interacting with the object. Food Object vs. Feeding: Determined by whether the elephant must interact with the food-delivery object to get food (e.g., hay net) or whether food is available without interaction with the object. MODIFIER: Food-Delivery Object
Feeding/Drinking	Acquiring/gathering and consuming (putting in the mouth) any food item without interacting with a food-delivery object. Drinking water, not bathing. Feeding: Requires the elephant to put something in its mouth; manipulating alone and chewing alone is not sufficient. LEVEL 2: Activity level (stationary, locomoting) MODIFIER: Food/water source
Enrichment (non-food) Object Interaction	Interacting objects that do not provision food and are not permanent (e.g., firehose ball). MODIFIER: Enrichment Object
Habitat Feature Interaction	Interacting with features that are permanent in the habitat (e.g., dusting, bathing, digging, rubbing body, climbing on logs, investigating features with feet or mouth). MODIFIER: Habitat Feature
Repetitive Behaviors (Stereotypy)	Motor: Repeatedly performing a behavior for 3 or more consecutive repetitions without interruption (e.g., route tracing). Non motor: Performing the behavior for 5 s or longer. Stereotypy: Determined by lack of purpose. If a behavior appears to have a purpose (e.g., scooping small food items in a repetitive manner), it may fit in a different category LEVEL 2: Major form of repetitive behavior
Locomotion	Walking or running (fast walking) more than 2 body lengths in any direction without stopping for 3 s or longer.
Stationary	Any stationary state without engaging in another behavior for 3 s or longer. Standing, walking or shuffling (without moving 2 body lengths), sitting, kneeling, lying (prone or sternal). LEVEL 2: Stationary body position (upright, lay down) MODIFIER: Location

**Table A2:** LEVEL 2: Social behaviors.

Function	Behavior	Definition
Affiliative	Trunk to: head area, trunk, body, front legs, mammary glands	Extend trunk tip to (within 6 inch) or visibly touches with trunk. MODIFIER: Sender/Receiver
	Trunk rub	Move trunk back and forth over body of another elephant (not for purpose of taking food atop the body). MODIFIER: Sender/Receiver
	Trunk twine	Mutual wrapping of trunks. MODIFIER: Partner(s)
	Tail tuck	Grab tail with trunk and tuck under front leg. MODIFIER: Sender/Receiver
	Lean/Rub	Lean or rest against or rub against another elephant while standing, sitting, or lying (includes under another elephant). Behavior includes: one elephant lying entirely or partially under another (e.g., calf lying under mom/auntie, elephants lying against each other, one elephant standing partially over another laying down. MODIFIER: Partner(s)
	Nudge	Gentle head-to-head, head-to-body, body-to-body contact initiated by one elephant towards another. MODIFIER: Sender/Receiver
	Greeting	Coming together, trunk tip to palatal pit (crease of mouth) with reciprocation. May include ear flapping, trunk entwining, arousal. MODIFIER: Partner(s)
	Play (Play contact, Play no contact, Play solicit, Self-play)	Play-contact: Sparring (pushing trunks, tusking, shoving, wrestling), back and forth nudging, trunk wrestling, chasing, rolling, climbing on conspecific (at least one foot on the head or body of another elephant). Play-no contact: Clear play with a conspecific (e.g., ball kicking, head wagging, splashing); play soliciting with approach and “run” away or other behaviors that seem to solicit play; other behaviors that seem like play but another elephant is not visible (e.g., sits on rump and throw back head, chasing wildlife, sliding on the mud). MODIFIER: Partner(s) or Unknown (no contact)
	Follow or Approach	Follow: Walk along path of conspecific with someone in the lead. Distance between animals almost constant. Approach: Move to within proximity (1 body length) of conspecific Sender = animal that is taking action—animal that is following or approaching. Receiver = animal being followed/approached Approach vs. Displace vs. Claim food/water/object: Depends on the sequence of events. MODIFIER: Sender/Receiver
	Share food/water	Simultaneously interacting with the same food-delivery object or feeding from the same food source, pushing food toward another, or drinking from the same water source. Requires that focal is proximate and has line of sight with partners. Behavior includes: Consuming food, sharing space around a food object even if unable to reach the object. Behavior does not include: Interacting with a food object when the elephants cannot see each other (e.g., on opposite sides of the enrichment tree) or bathing from the same water source. MODIFIER: Partner(s)
	Share enrichment object	Simultaneously manipulating same enrichment object. MODIFIER: Partner(s)
	Share habitat feature	Simultaneously interacting with the same habitat feature. MODIFIER: Partner(s)
Agonistic	Claim-food/object	Approach and take food or object w/in other elephant’s reach, on its body, or in its mouth. Includes displacing from a feeding/drinking source when the other elephant is still eating/drinking. MODIFIER: Sender/Receiver
	Displace-location	Approach and overtake position of conspecific, receiver moves at least 1 body length. Does not require physical contact. Behavior does not include: Pushing that results in displacement. MODIFIER: Sender/Receiver
	Block, resource hold	Place body between a resource and an approaching elephant. Holder may turn rump towards approaching elephant. MODIFIER: Sender/Receiver
	Strike or Push	Strike: Forceful body contact initiated by one elephant, including head butt (hit with forehead), trunk strike (hit with trunk), and kick (kick with any foot) Push: Forceful head-to-head, head-to-body, body-to-body contact that typically results in receiver being displaced, or at least it appears there is intent to displace or control. MODIFIER: Sender/Receiver/Partner (if mutual)
	Sparring	Mutual striking or pushing with head and trunk, tusking, driving. Sparring vs. Playing: Depends on the partner and appearance of aggression. MODIFIER: Partner
	Backup to	Backup rump first towards another individual’s head, side, or rump, sometimes extending tail to touch (moving towards another in a submissive manner). May end in a Rump Present. MODIFIER: Sender/Receiver
	Rump present	Back rump under the chin of another (submissive or reproductive, depending on partner and behavior sequence). MODIFIER: Sender/Receiver
	Tail pull	Hold tail with trunk and pull or twist. MODIFIER: Sender/Receiver
	Drive	Use head, tusks, or trunk to push the rear of another, maintaining contact while both elephants move. Drive vs. Pre-mount: Determined by whether the trunk of the sender is over the back of receiver with an attempt to get in mounting position. MODIFIER: Sender/Receiver
	Threat	Ears wide, trunk forward, head raised; may include foot scraping, twitching the tail, head shaking, or weaving. MODIFIER: Sender/Receiver
	Charge, mock	Rapidly approach another animal, ears erect, head high, tail extended, trunk sometimes extended. Stops short of contact. Often trumpets. Stops before contact, often in freeze position. MODIFIER: Sender/Receiver
	Charge, real	Rapidly approach another animal or object, ears usually close to head, head high, trunk tucked under head. Often silent. Attempts contact (strike, push). MODIFIER: Sender/Receiver
	Chase to retreat	Sender rapidly approaches receiver and receiver retreats. MODIFIER: Sender/Receiver
	Intervene	Place body between the sender and receiver in an agonistic interaction (typically by a dominant elephant).
Arousal	Spinning U/D–group	Spinning, urinating (U), defecating (D) as a group (usually in close proximity) or by one’s self with no contact. May include Arousal-other. MODIFIER: Partner(s), if any
	Pause/freeze, alert posture	Pause/freeze: No body movement, absolute stillness, typically with ears erect, sometimes w/ forefoot raised and still. Alert posture: Head raised, ears spread, tail raised, trunk raised.
	Defensive circle	Group forms defense circle around an individual MODIFIER: Partners
	Arousal-other	Foot scrape, tail erect (parallel to ground), tail up, trunk slap on ground; may include “running” (in the presence of other elephants). Does not include: “running” during play or with no social context or “running” with an enrichment object.
Chemosensory	Trunk to: vulva, penis, anus, back legs, temporal gland	Extend trunk to within 6 inch or visibly touches with trunk. MODIFIER: Sender/Receiver/Partner (if mutual)
	Trunk to: urine, feces	Extend trunk tip to (within 6 inch) or visibly touches with trunk the urine or feces, either from self or another elephant.
	Flehmen	Place dorsal trunk finger onto roof of mouth (VNO openings).
Reproductive	Rump present	Back rump under chin of another (reproductive or submissive depending on partner and behavior sequence). MODIFIER: Sender/Receiver
	Pre-mount Mount	Pre-mounting (chasing, driving with trunk over back) or mounting behaviors. MODIFIER: Sender/Receiver
Dam-calf	Nurse Attempted nurse	Nursing (dam stands for calf to suckle) or attempt nursing (dam does not move front leg forward or dam moves it leg back to block a teat).
	Claim calf	Secure space around the calf by physically moving the calf away from another elephant or blocking access to the calf. Appears like an act of protecting or owning.

**Table A3:** LEVEL 2: Activity level.

Activity Level	Definition
Locomotion	Feeding/drinking while locomoting
Stationary	Feeding/drinking while stationary

**Table A4:** LEVEL 2: Major form of repetitive behavior.

Major Form	Sub-Form	Definition and Examples
Whole-body stereotypy motor: 3 reps	Limb-swinging	Swinging the trunk or legs in a consistent pattern without body movement.
	Forward, backward, or vertical movement	Forward, backward, or vertical body movements along the axis parallel to the elephant’s spine. Does not involve travel. Rocking: Body and head move in unison in a forward/backward motion with little vertical movement of the head. Bobbing: Head moves in a vertical motion and may involve turning the chin from shoulder to shoulder. Bobbing typically corresponds with vertical elevation and depression of the spinal column between the shoulder blades and the pelvic girdle.
	Side to side movement	Body movement occurs along the axis horizontally perpendicular to the elephant’s spine. Swaying: Body and head move counter to each other, causing the body to undulate horizontally. Weaving: Body undulates side to side while the head is lifted or twisted vertically.
Locomotor stereotypy motor: 3 reps	Locomotor movement	Repeatedly walking the same pattern without leaving the path/route or without performing a competing behavior. Pacing: Following a relatively straight path in a forward or backward direction with patterned turns at each end of the path. Route tracing: Following a specific route. Route tracing patterns are more circuitous and longer than the straight paths associated with pacing.
Self-directed stereotypy motor: 3 reps non-motor: 5 s	Self-directed behavior	Self-stimulating behaviors. Limb or head banging: Striking one’s limb or head against objects in a non-species typical manner. Nipple pulling: Using the trunk to pull or manipulate one’s own nipples.
Oral stereotypy non-motor: 5 s	Oral behavior	Trunk sucking or “thumb sucking”: Sucking or chewing one’s own trunk Bar biting: Taking a bar into the mouth and biting or sucking on it. Chain chewing: Taking a chain or cable into the mouth and biting or sucking on it

**Table A5:** LEVEL 2: Stationary body position.

Position	Definition
Stationary state-Upright	Standing, waling/shuffling, sitting, kneeling.
Stationary state-Lay down	Lying (prone or sternal). Sternum or side is in contact with substrate; can vary from curled up to stretched out. Behavior does not include: Lying under another elephant, which is social

**Table A6:** MODIFIER: Stationary location.

Location
Gate or door
Under shade structure
Other location

**Table A7:** MODIFIER: Example resources in the previous and new habitats at the Oregon Zoo.

Food-Delivery Object	Enrichment Object	Habitat Feature
Timed feeder	Firehose ball	Pool—large
Enrichment tree	Buckets of snow	Pool—wading
Hanging hay net	Log, small	Large logs or rocks
Cement herd feeder with food	Tire, small	Tire, large
Spinner/pipe puzzle with food	Spinner/pipe puzzle	Cement herd feeders (to climb)
Boomer ball with food	Boomer ball	Habitat enclosure (walls, gates, doors, poles)
Barrel/keg with food	Barrel/keg	Substrate (dirt/mud, sand, gravel, straw, cedar shavings)
